# Towards an Integral Therapeutic Protocol for Breast Cancer Based upon the New H^+^-Centered Anticancer Paradigm of the Late Post-Warburg Era

**DOI:** 10.3390/ijms21207475

**Published:** 2020-10-10

**Authors:** Salvador Harguindey, Khalid Alfarouk, Julián Polo Orozco, Stefano Fais, Jesús Devesa

**Affiliations:** 1 Department of Oncology, Institute of Clinical Biology and Metabolism, 01004 Vitoria, Spain; polorozco@gmail.com; 2 Department of Pharmacology, Al-Ghad International Colleges for Applied Medical Sciences, Al-Madinah Al-Munawarah 42316, Saudi Arabia and Alfarouk Biomedical Research LLC, Tampa, FL 33617, USA; Alfarouk@hala-alfarouk.org; 3 Department of Oncology and Molecular Medicine, Istituto Superiore di Sanità (National Institute of Health), 00161 Rome, Italy; Stefano.fais@iss.it; 4 Scientific Direction, Foltra Medical Centre, 15886 Teo, Spain; jesus.devesa@usc.es

**Keywords:** breast cancer etiopathogenesis, breast cancer treatment, hydrogen ion dynamics of cancer, pH-related paradigm, H^+^-related therapeutics of breast cancer

## Abstract

A brand new approach to the understanding of breast cancer (BC) is urgently needed. In this contribution, the etiology, pathogenesis, and treatment of this disease is approached from the new pH-centric anticancer paradigm. Only this unitarian perspective, based upon the hydrogen ion (H^+^) dynamics of cancer, allows for the understanding and integration of the many dualisms, confusions, and paradoxes of the disease. The new H^+^-related, wide-ranging model can embrace, from a unique perspective, the many aspects of the disease and, at the same time, therapeutically interfere with most, if not all, of the hallmarks of cancer known to date. The pH-related armamentarium available for the treatment of BC reviewed here may be beneficial for all types and stages of the disease. In this vein, we have attempted a megasynthesis of traditional and new knowledge in the different areas of breast cancer research and treatment based upon the wide-ranging approach afforded by the hydrogen ion dynamics of cancer. The concerted utilization of the pH-related drugs that are available nowadays for the treatment of breast cancer is advanced.

## 1. Introduction

Breast cancer (BC) has become the second most prevalent cause of mortality in women [1]. Rarely does BC kill a patient from local disease—its morbidity is mainly secondary to a progressive and relentless metastatic process [2,3]. The results of surgery, traditional chemotherapy and radiation are often disappointing if not useless in advanced disease. Thus, a change towards a different perspective, incorporating more effective and less toxic approaches to treatment is highly necessary. Outside the cancer research community dedicated to cancer metabolism, the new anticancer paradigm based upon the pH/(H^+^)/proton dynamics is still a minority within mainstream anticancer approaches. The model described here considers all aspects of malignancy from one single and integral perspective in order to embrace a wide array of apparently unrelated factors involved in the etiopathogenesis of BC [4]. This approach, based upon the cancer-selective and deregulated proton (H^+^) dynamics in cancer, allows us to reach a new and deeper understanding of the intimate energetics of the acid–base dynamic nature of any malignant process, as well as of human neurodegenerative diseases (HNDDs), the latter as a beneficial side effect of the H^+^-related outreaching paradigm [4,5,6,7,8].

This new and wide-ranging perspective is able to absorb within itself most areas of cancer research. As a consequence of the selective acid–base homeostatic disruption and energetic failure of cellular hydrogen ion (H^+^) dynamics, attempts to induce significant intracellular acidification using proton transport and pump inhibitors (PTI and PPI), as well as other intracellular acidifiers and cancer proton reversal (CPR) agents of different origins and natures (repurposed drugs), are becoming an increasingly popular integral therapeutic strategy in cancer treatment [4,9]. The present contribution, from basic to translational to clinically oriented, for the first time, advances an integrated therapeutic approach describing the concerted and progressive utilization of a series of proton-related anti-cancer drugs that can already be used in bedside oncology in the treatment of BC. The new pH-based strategy allows us to interfere with all of cancer’s metabolic hallmarks, from prevention to the treatment of advanced disease. From this perspective, our group has recently published two conceptual publications, one on the pH-related etiopathogenesis and treatment of brain tumors [10], and a second one on breast cancer [11].

### Otto Warburg Today: pH as the Missing Link

One hundred years ago, Otto Warburg considered the respiratory impairment of tumors and their aerobic glycolysis as the prime cause of cancer. This was later called the Warburg effect. In one of his early studies, Warburg and his coworkers looked at the effects of hydrogen ions, bicarbonate and glucose concentrations in anaerobic conditions. They observed that glycolysis increased up to twofold when cells were gassed with increasing CO_2_—while maintaining pH at about 7.5—and, on the other hand, that CO_2_ gassing led to the acidification of the solutions, which resulted in reduced glycolysis [12]. In this publication, Warburg’s interpretation of the role of pH was focused on a compensating effect of bicarbonate, mimicking conditions in arterial and venous blood. Later, in a talk given at the Rockefeller Foundation, Warburg stated (about glycolysis): “Special attention should be drawn to the remarkable influence of the bicarbonate...” [13]. Thus, Warburg was aware that the pH was an important parameter in maintaining glycolysis in his culture system, but was he aware of its significance in cancer metabolism? It seems that he was never aware of this, since he did not address the subject of the role of pH in glycolysis again in his later work [14,15,16]. Instead, he discovered the factor responsible for the hydrogen transfer capacity in respiration: the nucleotide NADP [17].

Very heated discussions followed in 1956, mainly between Otto Warburg and Sidney Weinhouse, on the role and meaning of glycolysis and its relationship to oxidative phosphorylation in the etiology of cancer [18]. Unfortunately, the pH/glycolysis/cancer relationships were completely absent from those discussions and disagreements. Since then, many different attempts and theories to explain the cause and role of the increased glycolysis of tumors have been and are still considered [19]. Nowadays, the historical limitations of past decades can be understood because the cause–effect relationships of pHi elevations in stimulating glycolysis were first considered during Warburg’s old age and mainly outside the cancer context [20,21,22,23,24,25,26,27]. Thus, Warburg did not have the chance to understand and/or rightly interpret the complex etiopathogenic and metabolic relationships between pHi/pHe, aerobic and anaerobic glycolysis and/or the H^+^-dynamics of cancer, cancer-selective CPR or the concerted strategy of cancer cells and tissues. For him, those mysteries were still hiding in the future and were very far removed from the way we understand them nowadays. They were made possible only decades later, thanks to our increasing knowledge of the role of proton extruders/and/or membrane cell-bound H^+^ transporters and pumps (PTs and PPs) in elevating pHi and decreasing pHe (so increasing CPR) in all cancer cells and tissues [4,10,28]. A few decades later, Proton emission tomography (PET) technology would bring a revival of interest in Warburg’s genial work and theories: serendipity at work.

We know that, during Warburg’s life, there were no real methods to determine pHi. Thus, the conclusion that can be reached is that Warburg probably took for granted that the cytoplasm of cancer cells was acidic because of their high production of lactic acid, while, nowadays, we know that this the complete opposite of the real situation [14,29]. These are the reasons that fully justify the fact that Warburg could not be right on what he considered to be “the prime cause of cancer “, namely aerobic glycolysis. Nowadays, everything appears to indicate that the prime cause of cancer is not the aerobic glycolysis of tumors and/or the respiratory impairment of cancer cells, as Warburg defended until his death, as all evidence seems to indicate that **the prime cause of cancer is the main factor, inducing aerobic glycolysis itself, namely the selective intracellular alkalization of cells in all tumors and leukemias** [4]. In the same vein, recent publications from at least three different groups have concluded that the Warburg phenomenon can be fully explained by the stimulatory effects of pHi elevations in glycolysis [4,30,31,32].

It was not until half a century after Warburg’s death that a microenvironmentally integrated strategy for cancer cells and tissues was first described [33]. At the same time, the dynamics of the hydrogen ion were advanced to act as the unitarian multidimensional factor able to embrace, from a new perspective, the intimate relationships of cancer cells’ carbohydrate metabolism with intracellular acid–base changes [34].

Finally, in a sentence that became the founding motto of the International Society for Proton Dynamics of Cancer (ISPDC), Otto Warburg once said: “We can only cure what we can understand first” [6]. Since then, the H^+^-centric paradigm has become the cutting-edge and main issue of the metabolic cancer research community. This has allowed us to understand many aspects of the different areas of cancer research, mainly since transmembrane proton transporters (PTs) and their inhibitors (PTIs) increasingly came into play. Thus, one hundred years later, we need to keep talking about the Warburg effect, in spite of the fact that it appears to be fully explained by pH changes and cellular H^+^-dynamics [4,19,28,29,30,31,32].

## 2. Breast Cancer. pH-Related Etiology and Pathogenesis. the Basic Approach

**Figure 1:**
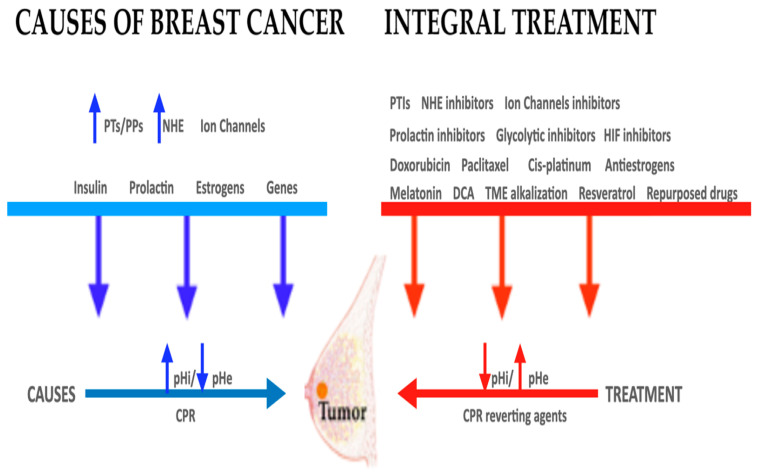
pH-related causes and available treatments for breast cancer. PTs: proton transporters; PPs: proton pumps: PTIs: proton transport inhibitors; PPIs: proton pump inhibitors; NHE: sodium–hydrogen exchanger; HIF: hypoxia-inducing factor; TME: tumor microenvironment; DCA: dichloroacetate. Blue arrows indicate causes of breast cancer. Red arrows indicate treatments of breast cancer.

Most of the discoveries and therapeutic proposals of the new H^+^-related paradigm have not yet entered mainstream bedside oncology, and, unfortunately, they still belong to the non-mainstream approach to cancer. In the meantime, the old, reductionist and almost exhausted anti-DNA model still dominates most areas of research and treatment in basic and clinical oncology. In order to overcome some of these limitations, we have attempted to integrate the most important etiological and pathogenic factors of BC from the all-inclusive pH-related perspective (Figure 1).

In spite of the fact that all proton extruders are different, they all share the same or similar effects on the etiopathogenic deregulation of the pHi/pHe (intracellular pH/extracellular pH) dynamics of cancer cells and tissues. These are described in the following subsections.

### 2.1. pH/NHE, H^+^ Extrusion and/or Intracellular Alkalization (CPR)

H^+^ extrusion from cells is induced by several membrane-bound proton transporters (PTs), pumps (PPs) and ion channels [4,35]. In cancer cells of all types and origins, they manage to keep pHi normal to elevated under all hostile tumor microenvironmental (TME) conditions, like low O_2_, anoxia, a lack of nutrients, low glucose levels and extracellular acidosis, mainly in order to protect themselves from a low pHi that would induce a cancer-selective therapeutic apoptosis. On the one hand, in 2000, Reshkin et al. demonstrated that, when the Human Papilloma Virus (HPV) transfected normal cells, the first protein that over-expressed was sodium–hydrogen exchanger 1 (NHE1) [36,37] and the pHi became more alkaline due to such NHE1 over-expression. On the other hand, extracellular signal-regulated kinase (ERK) is one of the proteins that phosphorylates and then activates NHE1 activity. The intracellular pH is a suitable medium for such a biochemical reaction [38,39].

The pHi alkalinity results in the upregulation of the utilization of glucose (Glycolysis + Pentose Phosphate Pathway), while it slows down the Krebs cycle [7,28]. Therefore, NHE1 over-expression reprograms the metabolic cell machinery to undergo Warburg metabolism and produce lactate, which is translocated extracellularly, creating an interstitial acidity that stimulates the already diseased tumor milieu [40]. After that, the acid pHe recruits the pro-inflammatory immune cells with their cytokines, further supporting the invasiveness process, diminishing the efficacy of many chemotherapeutic agents, stimulating proteases and fostering the metastatic transformation cascade [41,42]. In conclusion, the aberrant expression of NHE1 is a key determinant factor that drives a relentless progression and the integral neo-strategy of cancer cells and tissues [4].

**Figure 2:**
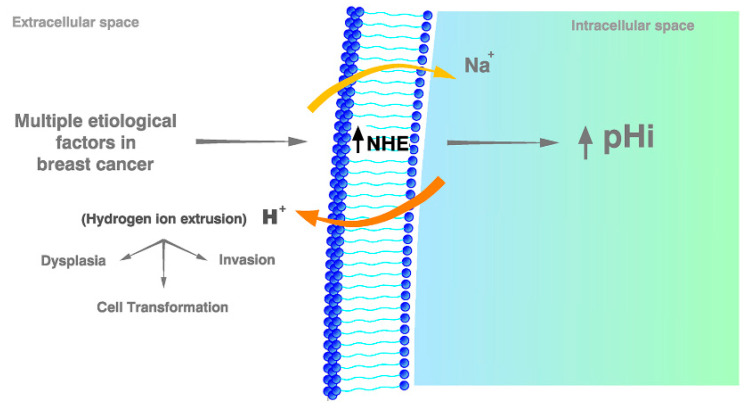
H^+^-extrusion in the etiology of breast cancer. Multiple etiological factors of different natures and origins are carcinogenic in breast cancer due to their positive regulation of NHE1 and/or intracellular alkalosis and/or extrusion of H^+^. This mechanism induces cell transformation and invasion (for further details, see text).

One of the most meaningful discoveries on BC etiology has been that H^+^ efflux alone induces dysplasia and stimulates growth and invasion with oncogene *RAS*, while inhibiting it induces apoptosis in invasive BC cells [43]. Most importantly, a series of publications in this line have shown that the H^+^ extrusion in BC cells, especially in triple-negative breast cancer (TNBC), is mainly mediated by NHE1 overexpression and H^+^-extrusion, with NHE1 being the real actor that induces a high pHi-mediated carcinogenic effect on breast cells [44]. Furthermore, H^+^ extrusion also is the main etiological mediator in the transition from ductal carcinoma in situ to invasive BC, where even a precancerous lesion already shows a higher than normal proton export rate [44,45,46] (Figure 2). Most recently, the intricate relationships of tumor pHi and pHe with ion channels and changes in membrane potential have been widely discussed and reviewed [47].

Furthermore, invasive BC cells show a more elevated pHi and higher production and exportation rates of hydrogen ions to the TME than noninvasive cells [44,48]. Most recently, however, other channels, transporters and even certain aquaporins (AQP), mainly AQP1 and AP3, as well as Cl^−^ channels with Ca^2+^ influx, have also been shown to be important in the initiation and progression of TNBC [49,50]. All these fundamental data clarify why and how NHE1, and its main consequences, H^+^ extrusion and an elevated pHi, are involved in BC etiology, invasion, and the metastatic process, as well as in multiple drug resistance (MDR) [43,44,45,46,51,52]. While a pathologically elevated pHi should be considered to be the main responsible factor of BC-promoting activity, it secondarily induces a highly pathological and damaging extracellular/intratumoral TME with the final result of inducing CPR. This selective and pathognomonic CPR has already become a highly differential hallmark of all cancer cells and tissues of all malignant tumors compared to normality [53,54]. This makes CPR reversal the main therapeutic and etiological target of the entire pH and/or H^+^-centric paradigm [4].

The cohort of all these new discoveries in the cancer context now contribute to making it possible to apply them to the highly specific molecular, biochemical, and metabolic abnormalities in the etiology and pathogenesis of BC. NHE1, NBCn1 (Bicarbonate-Dependent transport inhibitors), carbonic anhydrases (CAs) and monocarboxylate transporters (MCTs), mainly MCT4, are overexpressed in human BC, promoting the growth of at least triple-negative BC [55]. In this vein, NHE1 and NBCn1 drive cell cycle progression in human BC cells, while knocking them down reduces proliferation and tumor progression [56]. Finally, the activity of a significant number of carcinogenic and proangiogenic factors and oncogenes, as well as many other carcinogens, also upregulate NHE1.

Finally, NHE1 hyperactivity has recently been shown to be involved not only in the onset of cancer all along the digestive tract, from the degeneration of Barret’s esophagus into esophageal cancer to the relationship of inflammatory bowel disease and colon cancer, but also in the onset and promotion of atherosclerosis. This latter feature makes a hyperactive NHE also highly important outside the oncological setting [57,58,59].

### 2.2. Proton Transporters (PTs), Proton Pumps (PPs), and Growth Factors GFs)

NHE1 levels are higher in BC tissue than in normal breast tissue, and also in resistant BC cells than in sensitive cells, in a similar fashion to other PTs [4,10,51,55,56,60,61,62,63,64,65,66,67,68,69,70,71,72,73,74]. Apart from NHE1 overexpression, carbonic anhydrases also have an important role in the pathogenesis of BC, like V-ATPase proton pumps, the Na^+^–HCO_3_^−^ cotransporter (SLC4A7,NBCn1, MCTs, hypoxia and hypoxia-inducing factor 1 (HIF-1) [75]. A similar protumoral effect has certain oncogenes, gene mutations and products like *BRCA1* and *BRCA2*, apart from a dysfunctional p53 [76] and certain chemicals known to play a role in carcinogenesis [77,78]. These myriad factors belong to an already large list of mediating causes of cancer and BC previously reported [10,11]. This large amount of data demonstrates that many different etiological factors of different natures and origins all act through pHi/pHe dysregulation in the same direction, exerting a carcinogenic effect on BC pathogenesis.

### 2.3. Carbonic Anhydrases (CAs)

Membrane-bound carbonic anhydrases (CAs), mainly the isoform CAIX, have an important role in the pathogenesis of BC as well as in other tumors, especially in hypoxic conditions [65]. Being induced by hypoxia and HIF-1, CAIX overexpression is a sign of poor prognosis and promotes BC invasion and invasion in hypoxic microenvironments [79,80,81,82] Furthermore, CAIX expression, as well as NHE activity, are associated with estrogen receptor negative (ER^−^) BC tumors and a poor prognosis [11,83,84,85].

### 2.4. Monocarboxylate Transporters (MCTs)

In the same vein and with similar acid–base effects on cell homeostatic mechanisms to other PTs, MCTs induce: (a) further alkalization of the pHi of cancer cells; (b) worsening of TME acidosis by removing lactic acid from the intracellular space [55], and (c) cell proliferation, migration, invasion, angiogenesis and survival [86]. The isoforms MCT4 and MCT1 are the most significant and their overexpression is associated with tumor cell aggressiveness and a significant worsening of prognosis in either BC or other tumors [65,87,88].

### 2.5. The Sodium Bicarbonate Cotransporter (NBCn1)

The Na^+^–HCO3 cotransporter (SLC4A7, NBCn1) has also been considered to be the main mechanism of H^+^ extrusion, pHi elevation and CPR in BC, being actively involved in both BC carcinogenesis and in metastatic disease [54,61,89]. Indeed, its inhibition decreases BC growth rates and increases survival in mice by maintaining a sufficiently high pHi compatible with the growth and survival of BC cells [61,90]. Moreover, NBCn1 is synergistically associated with NHE1 and voltage-gated sodium channels like NaVi.5 [56,91,92]; while targeting Na(v)1.5 sodium channels, and also Ca^2+^ channels, it also diminishes invasion in metastatic BC [47,93,94].

### 2.6. Vacuolar ATPases (V-ATPase)

Since Peter Mitchell’s seminal description of the chemiosmotic hypothesis, this main energy-yielding mechanism dealing with electron transport and ATP synthesis in nature has also been considered as another acid–base mechanism induced by a H^+^ gradient across the mitochondrial membrane, a phenomenon dependent of ATPases [95]. Following this, a long period of time elapsed before BC could also be considered a molecular, biochemical and metabolic disease of an intimate acid–base nature [11]. As generally happens with PTs, V-ATPase proton pumps (PPs) are highly expressed in many tumors, apart from in BC, following the same rule of PTs, namely that their upregulation is a sign of bad prognosis, facilitating faster cancer growth, tissue invasion, the metastatic process and chemotherapy resistance (MDR), either in BC or other malignant tumors [96,97,98]. Moreover, the V-ATPase isoform a3 is selectively upregulated in BC cell invasion compared to noninvasive cancer cells and normal breast tissue [96].

V-ATPase over-expression offers a growth advantage to cancer cells of any origin, disrupting pH homeostasis in the same direction as PTs, while inducing a more abnormal CPR, that is, further increasing pHi, decreasing the tumor microenvironment pHe and, at the same time, acidifying endosomes and other intracellular organelles [73,99]. For all the above-mentioned reasons, V-ATPases have become very significant targets in any phase and in subsets of BC management. However, the main obstacle in treating cancer with V-ATPase inhibitors is that certain V-ATPases are ubiquitous in the human organism and, while they proved to be very active in in vitro conditions, they were shown to be highly toxic for normal cells in in vivo conditions. Therefore, drugs like Bafilomycin were abandoned as potential anti-cancer drugs [100].

### 2.7. Voltage-Gated Sodium Channels (VGSC) and Ca^++^ Signaling

Ion channels (ICs) are highly important membrane-bound additional factors in the etiopathogenesis of both cancer and human neurodegenerative diseases (HNDDs), pathologies that, from an acid–base point of view, dwell at both ends of a metabolic, biochemical and molecular spectrum [4,5,6]. In the cancer context, the most selective VGSC isoform is the NaV1.5-Na^+^ channel, which is synergistically associated with NHE1, both being overexpressed in BC and other tumors. Together, they promote local growth through invadopodia formation and stimulation of the metastatic process in a similar way as other PTs and PPs [91,92,101,102,103,104,105]. VGSC are expressed in highly metastatic cancer cells and are responsible for a sustained inward sodium current, H^+^ extrusion, membrane depolarization, TME acidification and an increase in the degrees of CPR [49]. Through these mechanisms, the invasiveness of cancer cells is enhanced by favoring the pH-dependent activity of acid proteases, cathepsins and pericellular tissue destruction [106]. ICs are especially important in the onset and progression of TNBC [107,108], as well as in its treatment [109]. Apart from ICs, Ca2^+^ signaling induces a potent oncogenic drive in BC [94]. This uniporter channel promotes triple-negative BC invasion and metastasis by favoring the Warburg effect [110].

### 2.8. Tumor Microenvironmental (TME) Acidosis

Apart from cancer-specific intracellular alkalization, the protumoral effects of the acidification of the TME are the second main metabolic and molecular issue in cancer growth and dissemination, either in BC and/or other human malignant tumors [111]. Its damaging effects are several: (a) locally stimulating tissue invasion and destruction, mainly by increasing the effects of invadopodia; (b) systematically disrupting the immune defense mechanisms of the organism and, in this way, fostering uncontrolled tumor progression; and (c) increasing MDR to most chemotherapeutic agents. Many other factors of different natures contribute to the pathogenesis, growth and spread of BC through TME acidification, such as hormones like estrogens, insulin, prolactin and sex steroids, growth factors like IGF1, EGF, VEGF and PDGF, as well as ion channels, cytokines and certain interleukins, apart from genetic abnormalities. Most of these factors, if not all, upregulate NHE1 [42,97,112,113,114,115,116,117,118,119,120,121,122,123,124,125,126,127,128,129,130,131,132,133,134,135,136]. Recently, a series of reviews covering the causes and consequences of tumor acidosis in cancer have been made available [118].

### 2.9. Estrogens

Estrogens are important in the genesis of BC. They promote cellular proliferation, while hindering apoptosis [83]. Estrogen-positive (ER^+^) cells are mainly associated with carbonic CAXII while CAIX is more frequently associated with estrogen-negative (ER^−^) cells [137,138]. Apart from BC, ER^−^ cells are characterized not only by a high expression of NHE but also of hypoxia-inducible factor 1 (HIF-1) [67,68,69,79,139,140]. Therefore, ER^+^ expression has been used not only as a prognostic indicator but also as a factor to be taken into account in the choice of BC treatment, perhaps with the exception of inflammatory BC [141].

### 2.10. Insulin (INS) and Insulin Resistance

The tumor-stimulating properties of insulin (INS) in BC are secondary to the fact that insulin stimulates NHE1, raising pHi and increasing glycolysis [28,142,143]. High glucose loads, with or without insulin, also stimulate Na^+^/H^+^ activity, cell cycle progression and the activation of oncogene expression [144]. These effects are associated with BC carcinogenicity and progression [11], justifying the fact that hyperinsulinemia and obesity are protumoral factors that increase the incidence of BC [145,146,147]. On the contrary, antidiabetic drugs of the sulfonylurea family, known to stimulate the pancreatic secretion of INS, appear to have a negative impact on BC growth, also increasing BC risk [148,149,150]. Furthermore, the overexpression of insulin and/or the insulin growth factor 1 gene are associated with a decrease in the length of the life of women with BC, while their suppression increases life span and decreases tumorigenesis [28,103,142,143,145,146,147,151,152,153,154,155,156,157]. Hyperinsulinemia has also been considered an important factor in a wide array of human malignancies, while insulin inhibition has been proposed to decrease their growth [158,159]. Furthermore, recent studies have supported the association of the insulin/IGF axis with cancer recurrence, including BC and colorectal cancer [150,160].

### 2.11. Prolactin (PRL)

The role of prolactin in stimulating the growth of BC, even as an etiological factor, is well established. Indeed, prolactin (PRL) stimulates local growth and the invasion of BC through NHE activation, in this way contributing to the metastatic process [161]. Recent studies have established that PRL signaling induces peripheral ruffle-targeted activation of NHE1 in BC cells [162]. Thus, PRL-mediated invasiveness of BC cells is NHE1 dependent, just with other hormones and growth factors (Figure 1). The development of BC also implies the association of PRL with other hormonal factors, like progesterone (PRG) and estrogen, whose chronic exposure also leads to hyperprolactinemia [163]. Furthermore, the interplay between PRL and progesterone in BC affects gene expression, producing a wider array of transcriptional regulators than those existing in the normal mammary gland. Finally, PRL, either coming from an increased circulating hormone or PRL produced by the mammary gland, have been found to induce BC in mice through the activation of the PRL receptor [164].

### 2.12. Genetic Abnormalities

The Na^+^/H^+^ exchanger isoform 1 (NHE1), as a fundamental factor in the etiology and pathogenesis of BC [43,44,45,46,51], is produced by the *APNH* gene located on chromosome 1 p35–36, which is also related to the etiopathogenesis of different tumors [165]. Other genes have a role in BC metastasis, at least 133, as well as 113 migratory modulators of Hs578T and MDA-MB-231 cells, which predict BC progression and carry a bad prognosis [154]. Moreover, *BRCA1* and *BRCA2* are associated with familial breast and ovarian cancers [166]. The possibility that the *BRCAs*’ carcinogenetic expression may also be secondary to NHE1 hyperactivity has been recently proposed [11].

### 2.13. MDR in Breast Cancer: Pathogenetic Mechanisms

The drug resistance of BC cells to drugs like doxorubicin (DOXO), paclitaxel and cis-platinum (CDDP) depend on pH regulation [167,168,169]. P-glycoprotein (P-gp) has been shown to need an H^+^ gradient in order to function [170,171]. Initially, cell studies showed that resistance to DOXO and P-glycoprotein were directly related, with drug resistance increasing with pHi elevations ranging from one at pHi 6.9 to more than 1000-fold at pHi 7.4 [172]. These important findings concluded that P-gp behaves as a proton (H^+^) extrusion pump [97]. A confirmation of this seminal information initially came from studies showing that the expression of P-gp leads to the elevation of pHi [130,173,174]. Furthermore, the levels of NHE1 are significantly higher in BC and other tumors when compared to adjacent normal tissues [175]. Moreover, there is an important role for V-ATPases in tumor invasion and chemoresistance in several cancers, including BC [73,176]. In summary, pH alterations have been shown to be behind the most fundamental aspects of MDR [97,125,177,178,179]. Finally, based upon the selective H^+^-dynamics of cancer, an integrated mechanism to explain MDR has been developed [41,97,125,127,128,167,168,170]. Unfortunately, all this important perspective and knowledge has been completely obviated so far by traditional bedside oncology practice.

## 3. Towards an Etiology-Based and H^+^-Related Integral Treatment for Breast Cancer: A Translational Approach

### 3.1. Intracellular Acidifiers (Proton Therapy): Na^+^/H^+^ Antiporter (NHE) and Other Proton Transport Inhibitors (PTI), Old and New

The main therapeutic target of the pH-centric anticancer paradigm is addressed to the concerted inhibition of NHE1 and other PTs and PPs to selectively induce an intracellular acidification (IA) incompatible with the life of cancer cells [4,101,180,181,182] (Figure 1). This attempt should also lead towards the control and even inversion of the tumoral extracellular/microenvironmental acidification of cancer tissues (TME). Tumor cell proliferation is further abolished through the concerted inhibition of NHE1 and HCO_3_^−^/Cl^−^ exchangers [183]. Similarly, the simultaneous inhibition of NHE1 and H^+^-ATPases induces cancer cell apoptosis through a combination of synergisms that lower intracellular pH (“metabolic proton therapy”) together with TME alkalization (“metabolic antiproton therapy”) [4,11].

### 3.2. On Amiloride (AM): Past, Present, and Future

In 1961, mitotic stimulation was considered to be secondary to membrane potential aberrations and electrophysical abnormalities. However, not a single mention of pH was made at that time [184]. This effect later was interpreted as being secondary to an increase in the intracellular content of sodium, but not to any pH changes [185]. Even though this conclusion survived the test of time [186], since the discovery of the Na^+^/H^+^ antiporter (NHE) [187], the emphasis on cancer initiation and etiopathogenesis turned from Na^+^ influx to its main consequence, namely H^+^ extrusion (Figure 2).

Amiloride (AM), a well-known diuretic and a weak and non-specific NHE inhibitor, has been commercially available for a long time and was the first Na^+^/H^+^ antiporter inhibitor used as an anticancer drug [188]. Treatment with AM completely inhibits the formation of lung metastasis in BC in rats [189,190]. Moreover, AM has been repeatedly reported to have antitumoral, antiangiogenic and antimetastatic effects [191,192,193], decreasing VEGF expression and inducing tumor growth inhibition through a significant decrease in pHi, at least in gastric and leukemic cells [188,194]. The many anticancer effects of AM on basic cell behavior have also been fully described [195]. The case of a patient who went into complete remission of metastatic ovarian cancer after being chronically treated with 15–30 mg/day of AM for one and a half years, after mainstream chemotherapy had failed, was reported [196]. With all these data available, it becomes difficult to understand why AM and other more potent and selective NHE inhibitors (Table 1) have not been considered at all in BC treatment, from prevention, in association with traditional chemotherapy, or as antimetastatic agents. Furthermore, two simple factors must be noted: (a) AM is very cheap and (b) it is not patentable. Moreover, a liposomal preparation of AM is commercially available. Finally, a wide array of these inhibitors and other antiangiogenic drugs are known to inhibit NHE1 [155,197].

### 3.3. Carbonic Anhydrase (CA) Inhibitors

Carbonic anhydrase (CA) is a family of several isoforms of the metalloenzyme CA, such as the cytosolic CAII and the transmembrane CAIX/XII, which efficiently catalyze CO_2_ hydration to bicarbonate and protons. By the coupling of these effects, a slightly alkaline intracellular pH is achieved (of around 7.2) at the same time as an acidic extracellular pH of the tumor is generated, with values as low as 6.5. Between the various CAs, CAIX and CAXII have been shown to have a prominent role in the regulation of tumor pH. Among them, CAIX has the most interesting features as a potential target of anti-cancer therapies [198]. Indeed, CAIX acidifies the TME under low-O_2_ conditions through HIF activity, promoting tumor cell survival and invasion in hypoxic microenvironments. In mice with BC treated with CAIX-specific inhibitors, there is a significant inhibition of tumor growth and metastatic formation, demonstrating that CAIX is fundamental for BC and should be used as a specific target in this disease alongside other PTI and PPI [4,11,80]. Interestingly, the combination of CAIX inhibitors and PPI has been shown to have synergistic antitumor effects [199].

Among the large number of sulfonamide, sulfamate, sulfamide, coumarin and CAIX/XII inhibitors reported to date, few compounds have been investigated in detail in animal tumor models, and only one of these derivatives, SLC-0111 (also known as WBI-5111), has progressed to clinical trials [200]. SLC-0111 also sensitizes cancer cells to conventional chemotherapy [201]. Interestingly, metastatic formation is inhibited in a T4 murine BC model by these novel CA inhibitors when used alone or with paclitaxel or doxorubicin [80].

Acetazolamide (AZM) is a CA pan-inhibitor and the only commercially available inhibitor and intracellular acidifier [11,68,82]. However, there are different prospective phase I/II studies with other more selective and powerful CA inhibitors, either being tested as anticancer drugs or in association with other, more conventional treatments. However, none of them has yet reached the clinical stage [67,140,175,201,202]. Since CAIX inhibition significantly reduces the invasion of BC cells, AZM represents a complementary drug that should be included in any integral treatment of BC, mainly in combination with other cellular acidifiers and PTI [67,69,79,81,82,140,175,202,203]. Finally, topiramate also inhibits CAIV and induces pHi acidification, at least in glioblastoma multiforme [11,204].

### 3.4. Monocarboxylate Transporter (MCT) Inhibitors

Quercetin is a weak pan-monocarboxylate transporter (MCT), inhibitor and intracellular acidifier that is commercially available in many countries [205]. Quercetin causes tumor regression by increasing apoptosis [206]. Its main role is to inhibit lactate extrusion from cancer cells by downregulating MCT1 and MCT4, in this way inhibiting growth by decreasing TME acidosis in BC as well as in a wide array of other malignant tumors [63,65,86,207,208]. MCT inhibition in BC cells in different conditions has confirmed the potential of lactate transport inhibition in BC treatment, which it also significantly decreases in in vivo tumor growth [86]. Until better and more specific MCT inhibitors are clinically available, quercetin should be incorporated into the integral treatment of BC along with other PTIs specialized in inhibiting H^+^ extrusion from cancer cells [209]. Since gastrointestinal absorption of this drug is very poor, the use of the liposomal drug form is advised [55,86,88,113,202,210,211]. Lonidamide is also an MCT inhibitor, but is no longer available in bedside oncology [101,211].

### 3.5. Bicarbonate-Dependent Transport Inhibitors (NBC1)

Since the expression of the electroneutral Na^+^–HCO3 cotransporter (SLC4A7, NBCn1) is upregulated in human BC and other malignancies, either in carcinogenesis or during the metastatic process, its inhibition becomes another therapeutic weapon that should be considered in BC treatment, while the cotransporter should also be assessed as an indirect TME alkalizer [212]. Unfortunately, there is no NBC1 inhibitor available that could be used in bedside oncology, since the ones known thus far, like trifolcin, DIDS (4,4’-diisothiocyanostilbene-2,2’-disulfonic acid) and nigericin, despite the pioneering work Tannock’s group, have been known to be too toxic to treat human cancer [213,214]. Fortunately, inhibiting CAs with a CA pan-inhibitor like AZM may also indirectly inhibit NBC1, at least partially [61]. Thus, any efforts to inhibit NBC1 should have an extra beneficial effect in that it is possible to integrate NBC1 into the concerted treatment of BC. In this regard, AZM, initially used to treat pain in advanced cancer patients, appears to have an antitumoral effect in the treatment of glioblastoma and in overcoming MDR, as well as in potentiating the effect of chemotherapy in other tumors [215,216,217,218]. Indeed, disrupting the Na(^+^)–HCO(3)(^−^) cotransporter NBCn1 decreases BC growth rates and increases survival in mice [61,90].

### 3.6. Proton Pumps/ATPAse Inhibitors (PPI), TME Alkalizers in Cancer Treatment, MDR, Cancer Pain and Tumor Immunity (Antiproton Therapy)

The clinical utilization of V-ATPase inhibitors of the omeprazole family (PPI) has an important therapeutic role in counteracting the highly pathological proton dynamics of BC and other tumors [11,99,219]. PPI are most effective in controlling the protumoral TME acidosis of tumors [35]. Recently, the use of anti-acidic drugs of the ATPase family as PPI has led to them being successfully exploited as anticancer agents in both pre-clinical and clinical conditions [100]. Different studies also support a direct anti-tumor effect of PPI independently of cancer histology [11]. PPI are also useful, together with CA inhibitors, in downregulating exosome production, which is known to be involved in the progression of different human malignancies [220]. Furthermore, another advantage of PPI is that they are prodrugs needing acidity for their full activation, thus lowering any side effects while being more effective in the acidified TME conditions of malignant tumors.

Other studies have used PPI, either as a single therapy or in combination with standard chemotherapy, in humans for BC with overall positive results, even when used in overcoming MDR [73,221,222]. BC patients receiving high PPI dosages obtained higher response rates and s longer survival [223]. Moreover, there is a significant increase in the survival of women who continue their PPI therapy after the completion of chemotherapy for BC [224]. On the other hand, women receiving PPI treatment for non-cancerous diseases have a reduced risk of developing BC [71,225]. Intermittent high dose PPI also improves MDR in metastatic BC [223]. Finally, V-ATPase inhibitors, along with other TME alkalizers, like acid-sensing ion channel 3, have been reported to improve lactic acid-mediated bone pain in metastatic disease in different human cancers [226,227,228,229,230,231,232].

TME acidity is known to blunt the immune defenses of the organism, which favors uncontrolled cancer progression and the metastatic process [114,120], since TME acidosis blocks T-cell activation [121]. Indeed, TME acidification has an essential role in the progression of inflammatory BC (IBC) [141], which makes TME a novel and fundamental therapeutic target in this most aggressive form of the disease. This therapeutic “antiproton therapy” should be continuously targeted in the chronic situation in BC in order to control, decrease and, if at all possible, revert TME acidity. To this end, large daily amounts of sodium bicarbonate plus dimethyl sulfoxide (DMSO (see Section 5.11 below) or other buffers have also been used in human cancer [230,231]. Thus, controlling TME acidity will correct T-cell dysfunction and allow for an improvement in the efficacy of any immunity-based anticancer therapies [116,117,122]. Therefore, we conclude that, for all these reasons, namely control of metastatic disease, pain therapy and immune failure, the TME has to be targeted in all types of BC patients within an integrated program of treatment, even from the earliest stages [119,233]. This can be done directly through TME buffering, and indirectly using PPI and PTI in order to decrease the lactate extrusion of cancer cells and collaborate in the induction of intracellular acidification (CPR reversal), which, ultimately, is the main and fundamental target of pH-related cancer therapeutics [4,234]. However, some serious concerns have also been raised in recent times regarding the possible negative effect of the indiscriminate use of PPI on cancer mortality [235].

### 3.7. Voltage-Gated Sodium Channel (VGSC) Inhibitors

VGSC, mainly Na(v)1.5 sodium channels, have become a relevant therapeutic target in cancer [157] since they promote cancer growth and invasion in BC [92,109,186]. Na(v)1.5 inhibition has been reported to increase survival in patients with BC [4,236]. Drugs like phenytoin, topiramate or ranolazine, as well as other repurposed drugs, can be used in decreasing invasion and metastases in BC by inhibiting Na(v)1.5 sodium channels. Thus, their utilization should be considered at least as complementary targets in any integral pH-related anticancer treatment [93]. The potential of ion channels in cancer has been extensively reviewed [237]. However, it is highly surprising that a clinical study of patients exposed to VGSC-inhibiting drugs has been associated with BC, bowel and prostate cancer patients [238].

## 4. Other pH-Related Available Therapies in Breast Cancer Treatment

### 4.1. Cisplatin (CDDP) and pH/NHE

Cisplatin has been used in the treatment of BC and other malignancies for a long time [239,240,241]. From its first introduction in bedside oncology, different mechanisms of action for CDDP have been described [242,243]. Until most recently, an almost completely disregarded issue has been the fact that cisplatin significantly changes the pHi of cancer cells, inducing cytoplasmatic acidification through the inhibition of H^+^ extrusion through NHE1 downregulation [241,242,244,245]. Indeed, this pHi-lowering effect has been considered to be the first effect of cisplatin on cancer cells [244]. Contrarily, the activity of NHE1 increases the resistance to cisplatin by elevating pHi [241,242,244,245], with this representing one more dualism of the pH paradigm. Apart from inducing pHi acidification, cisplatin shifts cells from glycolysis to oxidative metabolism. In this context, malignant cells either manage to maintain an alkaline pHi in order to survive and proliferate, or die [246].

### 4.2. Doxorubicin (DOXO) and Paclitaxel

Seminal studies showed that dynamic elevations of pHi induce a progressive increase in resistance to doxorubicin, at least in lung cancer cells, with this resistance being suppressed by P-gp inhibitors. Contrarily, P-gp increases pHi [172]. Furthermore, MDR is characterized by a selective reversal of the pH gradient (CPR) across all cancer cell membranes [4,10,97,167,168,172,174,179,202]. This allows for an understanding of why the concerted inhibition of NHE1 plus CA inhibitors improves the efficacy of paclitaxel by mediating its induction of apoptosis in triple-negative BC cells and its metastases [44,46,52,139,167,179,202,239,241,247,248]. More recently, liposomal preparations and nanodrugs of DOXO and others compounds have been trying to find a place in the treatment of BC and other tumors, and clinical trials with these methodologies are underway [249].

### 4.3. Antiestrogens

ER^−^ cells show a higher expression of NHE1 activity than ER^+^ cells, while CAIX is also more frequently associated with ER^−^ cells than with ER^+^ ones [137,138]. ER^−^ cells are also characterized by a higher expression of hypoxia-inducible factor (HIF) activity [11]. Thus, it can be understood that the selective inhibition of CAIX improves the prognosis of BC and that NHE1 inhibition is therapeutically indicated, at least in ER^−^ tumors [203] The roles of tamoxifen (TMX) and letrozole (LTRZ) are well established in the treatment of BC (see Section 5.5). Further connections among the pH paradigm and antiestrogens in BC have not been described, at least not directly [2,83,84,85,137,138,203,250,251].

### 4.4. Anti-Insulin Strategies and Metformin (MET)

Metformin has been introduced as an anticancer agent in BC bedside oncology. This antidiabetic drug has been shown to be an intracellular hyperacidifying agent in tumor models. It also functions as an anti-insulin factor, inhibiting insulin and insulin growth factor 1, while decreasing a wide array of other protumoral factors, like HIF-1α, Warburg metabolism, gene expression, angiogenesis, cancer migration, invasion and metastasis. It has been used to target resistant cells in BC and has even been proposed to be a radio-sensitizer agent [252,253,254,255,256,257,258,259,260,261,262,263,264,265,266,267,268,269,270,271,272]. Moreover, antidiabetic agents, like rosiglitazone and metformin (MET), show promising anticancer properties as INS-sensitizing agents [273,274,275], while sulfonylureas, because of their effects in stimulating INS release, have been considered to be pro-tumorigenic [276]. Finally, INS inhibition has been proposed as a complementary treatment in a small series of patients with advanced cancer [158]. In spite of the fact that INS deprivation has not yet been proved to be an effective therapeutic measure, a low-carbohydrate diet should always be contemplated in all BC patients in order to decrease the stimulatory effects of circulating INS on cancer growth, not least because high glucose loads induce intracellular alkalinity and the Warburg effect [277].

### 4.5. Prolactin (PRL) Inhibitors

NHE1 inhibitors decrease prolactin-induced BC invasion, while NHE1 activity is also decreased by the inhibition of Akt and/or ERK½, factors that are known participants in growth hormone (GH) signaling pathways, another hormone not to be forgotten in BC [162]. Furthermore, other antagonists of PRL/PRL receptor interaction are used in the treatment of BC, either alone or with tamoxifen (TMX) and/or letrozole (LTRZ) [161]. Disrupting the effect of PRL and/or PRL receptor expression delays oncogene-induced BC [278]. Thus, PRL inhibitors like the dopaminergic agonists bromocriptine and cabergoline should be taken into account as part of the armamentarium of drugs in BC integral therapy, even as drug sensitizers [279].

### 4.6. Melatonin (MT)

Melatonin (MT) has been postulated to be an antiestrogenic agent, so it should be strongly considered, at least in the treatment of the same cohorts of BC patients that can benefit from antiestrogens [280,281]. In BC cell lines, treatment with MT decreases tumor aggressiveness and increases apoptosis [282]. MT plays a number of other different actions as an anti-cancer agent [283]. Among them, it regulates the expression of estrogen receptors (ER), inhibits enzymes involved in the local synthesis of estrogens, activates the immune system and decreases angiogenesis by downregulating VEGF [280,284,285,286]. Finally, MT also inhibits other different angiogenic factors under hypoxic conditions [286].

MT has been reported to inhibit BC metastasis by maintaining a normal circadian expression of *BMAL-1* in tumor hypoxia-induced acidosis [287]. Another positive effect described for MT in BC is that it decreases the expression of both the glucose transporter GLUT-1 and Ki-67 (a marker of cell proliferation; therefore, its increase indicates a bad prognosis), while increasing the expression of the proapoptotic enzyme Caspase 3, therefore preventing the aggressive phenotype of BC cells under acidotic conditions [282]. MT also suppresses tumor aerobic metabolism (Warburg effect), inhibiting pathways that are key for the survival, growth and metastases of BC cells, while decreasing resistance to anti-cancer drugs [281,288,289,290]. Finally, since ER^+^ BC frequently develops genetic or epigenetic-induced resistance to antiestrogens [291], the new MT–TMX conjugates may represent a further improvement in the treatment of BC in these situations [292].

### 4.7. Repurposed Drugs

Dichloroacetate (DCA) is an anti-cancer agent that reverses the glycolytic phenotype in cancer cells by inhibiting pyruvate dehydrogenase kinase. Through this mechanism, the growth of several BC cell lines was found to be inhibited by DCA. This drug also shows anti-proliferative properties and pro-apoptotic properties, and can be effective against highly metastatic disease in vitro and in vivo [293]. DCA also improves immune dysfunction in different tumors [294]. Other repurposed drugs that have been reported to be active in BC because of their pHi-related acidifying effects are quercetin, resveratrol, phloretin, lonidamine, niclosamide, docosahexaenoic acid (DHA), simvastatin and the K^+^ ionophore salinomycin [295,296,297,298,299,300,301,302,303]. These and other repurposed drugs for cancer have been recently reviewed and proposed to show antiproliferative, pro-apoptotic and/or antimetastatic activity [304], either in BC or other tumors.

### 4.8. Overcoming Multiple Drug Resistance (MDR) in Breast Cancer: The Integral Approach

NHE1 inhibition and/or cellular acidification downregulate the MDR transporter [130,305,306]. Moreover, NHE is expressed in BC cells, mainly in ER– ones [307]. Thus, MDR and the CPR of cancer cells and tissues are related in a direct cause–effect relationship, as two phenomena that cannot be separated from each other [41,97,125,127,128,167,168,170]. Since extracellular acidification also increases the activity of P-gp, in this way inducing MDR in different cancer cells and tissues [125,128,132], it becomes logical to associate PPI with PTI, not only to improve the effect of chemotherapy in metastatic BC, but also to overcome MDR. The clinical use of such a combination is considered a fundamental therapeutic measure in any integrated clinical protocol in the treatment of BC [4,10,30,31,64,67,68,71,72,73,78,101,122,124,139,140,165,167,174,175,180,188,189,190,195,196,197,202,208,233,307,308,309,310,311]. The integral pH-related approach to MDR has shown that the therapeutic failure in inducing the acidification of the cytoplasm and/or reverse CPR is the main factor underlying MDR. In addition to a therapeutic cellular acidification, there are other mechanisms to restore sensitivity to CDDP, like targeting V-ATPase, impairing endosomal function and inhibiting autophagy [312]. It is concluded that MDR is systematically characterized by an inversion of the pH gradient (CPR) across cancer cell membranes [4,10,97,167,168,172,174,179,202], once more making CPR the main cancer-selective therapeutic target in any H^+^-related treatment of BC in bedside oncology.

### 4.9. Proton Therapy: Metabolic, Radiotherapeutic, or Both?

In previous sections (Section 3.1; Section 3.2; Section 3.6), proton therapy and antiproton therapy have been considered in the pH-related metabolic treatment of cancer. Recently, PT has become the latest and most advanced method in radiotherapy (RPT), either in the oncology setting or in other clinical situations [313]. However, we are not aware whether, among the mediating effects on cells and tissues that have been described for RPT, any changes in hydrogen (H^+^) concentrations or pH-related physical dynamics of the radiated tissues have been described or even considered at all [314].

### 4.10. H^+^-Related Autophagy in Cancer: The Coronavirus Connection

Autophagy has been extensively investigated in the treatment of cancer; however, its role remains elusive [315]. Moreover, after the formation of the initial autophagosome, it fuses with the other internal vacuoles with non-specific roles in the digestion of unwanted material, which makes it difficult to distinguish autophagy from the other phagocytic processes. Thus, if there is an important role of autophagy in cancer, it is still up for debate [316]. On the contrary, cell cannibalism and other cell-to-cell phenomena have been proven to present an active role in cancer [316].

Among other repurposed drugs, the antimalarials chloroquine and hydroxychloroquine have been studied in several clinical trials in oncology and are suggested to benefit certain cancer patients, at least in glioblastoma multiforme. This effect has been blamed on their effect in inhibiting autophagy [317,318]. As in other pH-related cancer treatments, the acidic pH of the TME neutralizes the uptake of CQ by tumors [319]. Like in cancer, V-ATPase plays a significant role in the degree of activity of the malaria parasite; however, their interrelationships are very complex [320,321]. The interest in these associations resides in the fact that CK and HQ have been initially studied—however, without success—in clinical trials during the COVID-19 pandemic [322] because they had been previously effective in other viral infections like SARS [323]. These data suggest that, both in COVID-19 and cancer, high doses of V-ATPase inhibitors of the omeprazole family can act like other, more powerful alkalizing agents like bleach and could be effective in overcoming resistance to CQ and/or HCQ in both cancer and COVID-19 infection.

### 4.11. Hypoxia-Inducible Factor (HIF) Inhibition

Tissue hypoxia on its own is an important factor in the etiology, physiopathology and development of different malignant tumors [195], mainly in scar tumors [195,324]. Furthermore, these prooncogenic situations can also be mediated by the hypoxia-inducible factor (HIF), which involves the transcription of a wide array of genes, allowing cancer cells to adapt, survive and grow in the most hostile hypoxic conditions [209], as well as inducing chemotherapy resistance [132,325]. In recent years, increasing efforts have been made to suppress HIF as an anticancer and antiangiogenic method to induce regressions in BC and other tumors [212,326,327]. To this end, a series of compounds and strategies were initially proposed and continue to be actively researched nowadays [207,266,269,286,328,329,330,331].

## 5. Towards a H^+^-Related Concerted Utilization of Clinically Available Drugs in the Integral Treatment of Breast Cancer: The Clinical Approach

### 5.1. Amiloride (AM)

Amiloride is the only NHE inhibitor commercially available nowadays. In cancer patients, it has been used at dosages of 10 mg, three times a day, continuously, over months or years. These dosages are well tolerated; however, some degree of hyperkalemia occasionally ensues (K^+^ up to 6 mmol/L), as well as increases in BUN (up to 90 mg/dL). In those cases, AM is discontinued for two weeks and restarted at a lower dose [182,191,192,196]. No other side effects have been found after its chronic utilization in a wide array and number of cancer patients.

### 5.2. Carbonic Anhydrase (CA) Inhibitors

Acetazolamide (AZM) is the only commercially available CA pan-inhibitor. It is used in the treatment of glaucoma and as a diuretic or in the treatment of epilepsy. Oral dosages for AZM range from 250 to 1000 mg/day. Like with amiloride, it is important to have blood tests for K^+^ and BUN every 3 to 4 weeks. AZM, as a CA pan-inhibitor and cell acidifier, represents a very promising drug in the treatment of BC, mainly in combination with NHE inhibitors. Although pre-clinical research has produced a list of potentially effective new CA inhibitors that are small molecules, mostly directed against CAIX, there is no further information regarding dosage and effects [198]. Other CAIX inhibitors, like SLC-0111, are indicated for hypoxic and acidic cancer cells that are chemotherapy-resistant. CA also increases BC cells’ response to doxorubicin [201].

### 5.3. V-ATPase Inhibitors and/or Proton Pump Inhibitors (PPI) (Antiproton Therapy)

While many pre-clinical in vivo studies have shown the efficacy of PPIs as single anti-tumor agents, they have been exclusively used in combination with traditional chemotherapy. Based on pre-clinical investigations, the initial treatment protocols are based on three rules: (1) they should be used before chemotherapy, due to the evidence that they are needed to abrogate tumor acidity in order to allow other drugs to fully work; (2) high dosages, between 1.5 and 2.5 mg/kg/day of lansoprazole or pantoprazole, are recommended. Lately, it has been shown: (a) that lansoprazole is the most active PPI; (b) that dosages change according to gender, i.e., 90 mg/day for men and 60 mg/day for women; and (3) that a continuous daily treatment is used for at least one year. On the other hand, an intermittent high dose of PPI also enhances the antitumor effects of chemotherapy in metastatic BC; however, intermittent high doses of PPI have been reported to enhance the antitumor effects of chemotherapy [223]. All these conclusions have been supported by a retrospective analysis in women receiving PPI for non-cancer-related ailments (i.e., gastroprotective or anti-acidic treatments, lansoprazole, 30 to 40 mg/day), showing that a gastroprotective dose is also adequate to protect against BC development [71,225].

### 5.4. MCT Inhibitors

Quercetin (QUER), or liposomal quercetin, is the only commercially available MCT inhibitor. Oral doses of three grams a day of QUER are well tolerated in the long term. However, since the oral absorption QUER is very low, the use of a liposomal form (LQUER) of the drug is advisable. Tolerance is excellent at doses of LQUER of 30 mg (concentration 1 mg/mL), three times a day.

### 5.5. Cisplatin (CDDP), Paclitaxel (PCXL) and Doxorubicin (DOXO)

Since many therapeutic protocols with different schedules and dosages of cisplatin/paclitaxel, cisplatin/doxorubicin, or paclitaxel/doxorubicin have been available for a long time in the treatment of the different stages and subsets of BC, either as neoadjuvant therapies, in early stages, or as treatments of advanced disease, no chemotherapy protocol will be considered here [332,333]. Furthermore, as this subject is outside the scope of this contribution, we are not aware that any of the many different chemotherapeutic regimes used in the treatment of BC have ever been associated with the pH dysregulation known to be fundamental in the treatment of BC or any other malignant tumors.

### 5.6. Antiestrogens

About 70% of women with BC show estrogen receptor (ER)-positive/HER2^−^ negative tumor cells. In these subsets, tamoxifen (TMX) has been widely used because it binds to ER, impeding the tumor- promoting action of estrogens. Oral dosages of TMX range from 20 to 40 mg, administered in one or two doses a day over at least five years. However, several adverse effects have been reported for this drug; among them an increased incidence of uterine cancer, presumably associated with its estrogenic effects on these tissues [334]. Moreover, an increased risk of thromboembolism has been reported during treatment with TMX or aromatase inhibitors [335].

Aromatase inhibitors lead to a decreased or absent production of estrogens by the adrenal glands. There are many aromatase inhibitors available, such as exemestane, anastrozole and letrozole, but none of them is free of toxicity; for instance, they produce osteoporosis or an increase in cholesterol levels [336]. Letrozole is used in postmenopausal women after five years of treatment with TMX at a daily dose of 2.5 mg, orally. Very recently, a phase II study conducted in Japan in 42 postmenopausal patients investigated the efficacy and safety of the combination of palbociclib plus letrozole, concluding that this combination is effective in patients with ER^+^, EGFR^−^ and advanced BC [337].

### 5.7. Antiglycolytic Drugs and Insulin Inhibitors

Targeting glycolysis has represented a promising theoretical approach to the metabolic management of cancer for many years. The interruption of glycolysis would interrupt the cytoplasmic utilization of glucose by cancer cells, inhibiting cell growth and invasion, while activating the Krebs cycle [338]. Drugs that decrease the cytoplasmatic utilization of glucose include 2-deoxyglucose, lonidamine, 3-bromopyruvate, imatinib, oxythiamine and hydroxycitrate, as well as other drugs that restore mitochondrial function, like alpha-lipoic acid [338,339]. Moreover, glycolysis has been reported to be paradoxically inhibited by the administration of buffering agents like sodium bicarbonate or potassium citrate [340,341].

### 5.8. Prolactin (PRL) Inhibition in Breast Cancer

Despite the significant role that PRL plays in BC and its invasiveness, plasma levels of PRL are not usually analyzed in BC or PRL inhibitors used in its treatment. Dopaminergic agonists are used for PRL pituitary adenomas. In BC, perhaps the best option would be to administer a dose of bromocriptine that is able to decrease circulating PRL to undetectable levels. In situations in which PRL values are over the normal range, bromocriptine is given at oral doses beginning at 2.5 mg, three times/day, but in the case of BC it is likely that higher doses should be contemplated. Bromocriptine has been developed as a long-acting injectable presentation (Parlodel-LAR*). Doses are in the range of 100 to 150 mg, intramuscular, every 4 weeks, but, thus far, its use is usually restricted to prolactinomas [342]. Cabergoline, a more recently discovered dopaminergic agonist, has the advantage of presenting longer lasting dopamine agonist effects. In fact, a single oral dose of 0.5 mg of cabergoline leads to a marked fall in plasma PRL for at least seven days [343].

### 5.9. Melatonin (MT) in Breast Cancer Treatment

In nurses or other female workers that work shifts, the prevalence of BC is higher than in the normal population, a feature that appears to be secondary to the disruption of the normal MT circadian rhythms [344,345]. Chronic treatment with MT is thought to have a preventive effect on BC incidence in these cases [346]. Indeed, the positive effects of MT on different cancers, particularly in BC, have been widely considered since 1992, when it was published that MT might be a natural oncostatic agent useful in BC prevention [347]. Despite the fact that doses of commercial presentations of MT are very low, in the range of 1.9–10 mg, MT can be used at much higher dosages without showing any adverse side effects. In fact, the toxicity of MT is very low, as many animal and human studies have demonstrated. In animals, a lethal dose for 50% of the animals (LD50) could not be found, and very high doses, such as 800 mg/kg body weight did not produce any adverse effects [348]. Similarly, no side effects were observed in a phase II clinical trial in which 1400 women were treated with 75 mg of MT daily, at night, for four years [349], nor were any adverse effects seen in a woman who took 50 mg of MT/daily for 9 years [350]. Moreover, 31 patients with Amyotrophic Lateral Sclerosis (ALS) were treated with rectal MT (300 mg/day) for 2 years without experiencing adverse effects from these high dosages [351]. Most recently, MT has been proposed as an antiglycolytic agent that inhibits Warburg-like metabolism and increases glucose oxidation [352].

Daily oral doses of MT in BC oscillate between 200 and 400 mg, every night. Locally, MT may also be used in the prevention of the side effects of chemo/radiotherapy, such as oral mucositis and dermatitis. In the case of mucositis, MT is given as an oral gel, applied by rinsing the mouth without swallowing it [353]. Moreover, an MT gel can be applied in the zone to be irradiated 15 min before each session to avoid the damaging effects of radiation on the skin [353,354].

### 5.10. Treating Tumor Hypoxia

Many HIF-1 inhibitors have been studied, but no selective HIF-1*α* inhibitor has been clinically approved. However, there are a few other drugs that can be used as complementary treatments for some types of cancers. This subject has been extensively reviewed in recent years [355]. In this vein, carbonic anhydrase inhibitors have been reported to suppress BC and other tumor growth and metastases by targeting hypoxia-induced CAIX [80,200,356].

### 5.11. Resveratrol 100 mg Capsules

Dosage: 100–1000 mg three times a day, permanently. Liposomes of resveratrol are also available.

### 5.12. Tumor Microenvironment (TME) Alkalization with Sodium Bicarbonate (SB) Plus Dimethyl Sulfoxide (DMSO) (SB^+^DMSO) in Cancer Treatment (Antiproton Therapy)

Alkalization with sodium bicarbonate (SB) alone has been shown to be effective in inhibiting metastases [119], while other methods of acid–base manipulation in the same direction have been reported to be clinically useful in treating intractable pain in cancer patients [357]. Most recently, systemic alkalization with small doses of sodium bicarbonate has been reported to improve the effects of chemotherapy in pancreatic cancer [358]. Moreover, recently, bone metastases from BC have been associated with TME acidification and lactic acid extrusion [135]. A mixture of dimethyl sulfoxide and sodium bicarbonate has been shown to be a safe and effective treatment for pain in advanced cancer patients [230,231,232,359]. It has also been reported that there is a prolongation of survival in advanced BC when using SB plus DMSO [231,360]. Finally, the utilization of DMSO in humans has demonstrated its lack of toxicity when used for periods of up to 5 years [360]. No increases in Na^+^, salt retention or in blood pressure are observed when the SB^+^DMSO mixture is used for months, or even years, and on a daily basis. However, Na^+^, K^+^ and BUN should be checked at least monthly during treatment. Unexpectedly, the alkalization of tumor pH and pHi with SB has shown cancer-promoting effects in BC tissues [361]. It is also paradoxical that the utilization of sodium bicarbonate, at least in certain circumstances, may lead to intracellular acidosis, with this phenomenon being more evident at higher starting intracellular pHs [362].

Formulation of the SB+DMSO mixture:

Thirty-four percent DMSO (99.9% pharmaceutical quality/99.9% purity), 64% double-distilled water and 2% SB.

Dosage: 10 mL orally, two to four times a day, on an empty stomach and separated from other medications. Only crystal bottles or high-density polyethylene (HDPE) should be used as containers, since DMSO can dissolve other kinds of plastic containers and become toxic to the patient. The simultaneous utilization of SB^+^DMSO along with any other chemotherapy protocol is also recommended.

### 5.13. Repurposed Drugs

Among a large list of repurposed drugs proposed for the treatment of BC and other tumors [4,100], salinomycin has been shown to induce partial regressions of several pretreated cancers [303]. Furthermore, treatment with DHA has been shown to increase survival in BC patients with metastasis. The daily doses used in these clinical trials were in the range of 1.800 mg DHA/day.

### 5.14. Bicarbonate Transporter Inhibitors (NBC1)

There is no specific NBC1 inhibitor available for clinical utilization. However, knockdown of NBC1 has prolonged tumor-free survival and reduced cell proliferation in basic studies through a pHi-lowering effect [55]. Finally, the utilization of different ion channels, transporter inhibitors and antagonists has been recently considered in different attempts to downregulate NBC1 [49].

## 6. Powerful NHE Inhibitors in the Treatment of Breast Cancer

### 6.1. Cariporide

Unfortunately, cariporide is not usually available for human use in bedside oncology because the patent holder decided to remove it from clinical trials in cardiology after some unexpected side effects were found. This was explained in the previous publications of our group [307]. However, this drug is available in a highly purified form from different sources around the world.

### 6.2. Compound 9t (C9t)

Compound 9t (C9t, a 5-aryl-4-(4-(5-methyl-1H-imidazol-4-yl) piperididn-1-yl) pyrimidine analog), is perhaps the most promising anticancer drug of the pH-related anticancer armamentarium, but it is not available for either basic or clinical research. Recently, the patent holder (Bristol Meyers Squibb) released the patent for the entire world, with the exception of the United States (until 2020) [307]. However, the description of the process of its synthesis was somewhat incomplete and, thus far, all efforts to synthesize C9t in different countries have been unsuccessful (patent: Bristol Meyers Squibb, WO 01 27,107 A2, PCT/US00/27, 2001,US 6,887,870 B1; EP 1,224,183 B1) [363]. C9t has been shown to be 500-fold more potent against NHE1 than cariporide. Furthermore, C9t is orally bioavailable, has low side effects in mice and presents an improved safety profile over other NHE1 inhibitors [307]. Compound 9t promises to act as a kind of “magic bullet-like” drug in a number of human malignancies.

### 6.3. Phx-3

Apparently, Phx-3 has been used in Japan for the treatment of inflammatory bowel disease [364].

## 7. Conclusions

In this contribution, the seminal acid–base aspects of cancer metabolism are considered from a fresh and integral perspective, starting with Otto Warburg’s highly significant discoveries and running into the long post-Warburg era. Mainly thanks to the discovery of PET technology, Warburg theories were resurrected and, despite their historical limitations, they have allowed a burst of new interest in cancer carbohydrate metabolism, along with its multiple basic, translational and clinical derivations. As a beneficial side effect of this growing evolution, a new pH-centric and/or H^+^-related paradigm was born and has rapidly evolved to give way to an entirely different perspective of the entire field of metabolic cancer research, far beyond the previous antiDNA paradigm of traditional oncology that has dominated cancer research and therapeutics during the last few decades.

From this new, wide-ranging “acid–base” approach to cancer molecular biology, biochemistry, and metabolism, most of the etiological and pathogenetic factors of human cancer can now be interpreted through a single and unitarian viewpoint. The cancer-specific combination of intracellular alkalization and its secondary extracellular acidification of all malignant tumors, which represents the mirror image of normality (acid inside/alkaline outside), confirms what has been defined as cancer proton reversal (CPR). This reversal of intracellular/extracellular proton dynamics is induced by the expression and/or upregulation of membrane-bound proton transporters (PT) and (PP) pumps, whose concerted etiopathogenic role, apart from preventing cancer-damaging cellular acidification by extruding H^+^ from the cell by all possible means, creates a series of progressive and strategic dynamic abnormalities. Indeed, these occur from the onset of the malignant process until the end of it, which, many times, leads to the death of the patient. For these reasons, PT and PP have become targets of the rapidly increasing therapeutic efforts in modern cancer research.

CPR itself has already become the primordial therapeutic target of all these efforts. The entire paradigm has grown to conclude the fact that the concerted utilization of proton transport inhibitors (PTI) and proton pump inhibitors (PPI), when used in pharmacological doses, could selectively decrease the pHi of cancer cells to apoptotic levels though a chain reaction-like mechanism, a concept that reminds us of the magic bullet dream of Paul Ehrlich’s theories. Moreover, the therapeutic alkalization of the tumor microenvironmental extracellular space (TME) appears to be the most practical and important measure that further contributes to the therapeutic reversal of CPR.

All of these conceptual and practical advances, as well as the increasing basic and clinical experience in metabolic cancer research, are integrated into this contribution, which is specifically dedicated to the pH-related etiopathogenesis and treatment of breast cancer from the new and integral perspective afforded by the hydrogen ion (H^+^)-related anticancer paradigm.

## Figures and Tables

**Table 1:** pH and/or H^+^-related options in the treatment of breast cancer. BC: breast cancer; NHE: Na^+^/H^+^ antiporter; P-gp: P-glycoprotein; MDR: multiple drug resistance; ER^+^: estrogen-positive cells; ER^−^: estrogen-negative cells; PTI: proton transport inhibitors; PPI; proton pump inhibitors. CAs: carbonic anhydrases; TME: tumor microenvironment. (For further details, see text).

Drugs	Effects
Amiloride (AM) (and/or liposomal amiloride)	AM is a non-specific and weak NHE inhibitor and cell acidifier. It also behaves as an antiangiogenic agent and has been proven to be able to completely abrogate the metastatic process in transplanted BC in rats.
Acetazolamide (AZM)	AZM acidifies cells by inhibiting certain carbonic anhydrases (CAs). In BC, AZM is effective in reducing tumor invasion. For an increasing clinical effect, AZM can be used together with NHE inhibitors.
Monocarboxylate transport (MCT) inhibitors	Quercetin is an MCT inhibitor and cell acidifier. Gastrointestinal absorption is limited. To overcome its scarce oral bioavailability, a liposomal preparation is available.
V-ATPase inhibitors (PPI) (antiproton therapy)	PPI are occasionally used in the prevention of BC and in overcoming MDR. PPI also benefit from the extracellular acidity of tumors. Recent clinical studies support the utilization of PPI in BC and other solid tumors.
Doxorubicin (DOXO)	pHi elevations are directly related to increasing resistance to DOXO. P-gp also increases pHi, while P-gp inhibitors decrease DOXO resistance.
Paclitaxel	NHE1 inhibition improves the effect of Paclitaxel in triple-negative BC metastasis. Paclitaxel has also been shown to induce apoptosis in BC cells.
Cisplatin (CDDP)	The first effect of CDDP consists of the induction of cellular acidification, inhibiting H^+^ extrusion through NHE1 downregulation. On the contrary, NHE-1 hyperactivity increases < CDDP resistance by elevating pHi.
Antiestrogens	ER^−^ BC cells correlate with a high expression of NHE activity and are also associated with CAIX upregulation. Estrogens and CAIX inhibition improve BC prognosis.
Melatonin (MT)	Because of the claimed antiestrogenic effects of MT, it should be contemplated in the integral approach to BC therapy. MT decreases tumor aggressiveness and increases apoptosis in BC cell lines. MT also suppresses tumor aerobic metabolism (the Warburg effect) and decreases BC angiogenesis and metastasis.
Metformin (MET)	MET induces intracellular hyperacidification in tumor xenograft models. It has been reported to inhibit insulin and IGF-1, HIF-1α, Warburg metabolism, gene expression, angiogenesis, cancer migration, invasion, and metastasis. It also decreases the side effects of doxorubicin. MET acts synergistically with chemotherapy and decreases its side effects.
Treating hypoxia-inducible factor (HIF) and tumor hypoxia	HIF activity promotes tumor cell survival and invasion. CAIX inhibitors have been reported to suppress BC growth and metastases by targeting tumor hypoxia and HIF. Different compounds and strategies have been tried to suppress HIF in BC research and treatment, so far without too much success.
Repurposed drugs	Because of their pH/related effects, drugs like dichloroacetate, phloretin, lonidamine, niclosamide, docosahexaenoic acid, salinomycin, simvastatin and resveratrol have been reported to be useful in the treatment of BC.
Cariporide (CP)	CP is a powerful NHE1 inhibitor that is barely available for clinical use in bedside oncology, although it can be purchased in a highly purified form from different sources. It induces apoptotic cell death in BC and other malignant tumors.
Compound 9t (C9t)	C9t is the most potent and selective NHE1 inhibitor. Furthermore, it is orally bioavailable, has low side effects in mice and it presents a significantly improved safety profile over other NHE1 inhibitors. Unfortunately, it is not available for preclinical or clinical research, apparently because of the complicated method of synthesis and purification.
Phx-3 (2-Aminophenoxazine-3-one)	Phx-3 is a potent, selective, and non-toxic NHE1 inhibitor that has been shown to be highly effective in animal tumor models. It has also been used in Japan to treat gastrointestinal inflammatory disease.

## References

[1] Evans D.G., Howell A. (2007). Breast cancer risk-assessment models. Breast Cancer Res..

[2] Early Breast Cancer Trialists’ Collaborative Group (EBCTCG) (2005). Effects of chemotherapy and hormonal therapy for early breast cancer on recurrence and 15-year survival: An overview of the randomised trials. Lancet.

[3] Egger S.J., Willson M.L., Morgan J., Walker H.S., Carrick S., Ghersi D., Wilcken N. (2017). Platinum-containing regimens for metastatic breast cancer. Cochrane Database Syst. Rev..

[4] Harguindey S., Stanciu D., Devesa J., Alfarouk K., Cardone R.A., Polo Orozco J.D., Devesa P., Rauch C., Orive G., Anitua E. (2017). Cellular acidification as a new approach to cancer treatment and to the understanding and therapeutics of neurodegenerative diseases. Semin. Cancer Biol..

[5] Harguindey S., Reshkin S.J., Orive G., Arranz J.L., Anitua E. (2007). Growth and trophic factors, pH and the Na^+^/H^+^ exchanger in Alzheimer’s disease, other neurodegenerative diseases and cancer: New therapeutic possibilities and potential dangers. Curr. Alzheimer Res..

[6] Harguindey S., Orive G., Cacabelos R., Hevia E.M., de Otazu R.D., Arranz J.L., Anitua E. (2008). An integral approach to the etiopathogenesis of human neurodegenerative diseases (HNDDs) and cancer. Possible therapeutic consequences within the frame of the trophic factor withdrawal syndrome (TFWS). Neuropsychiatr. Dis. Treat..

[7] Alfarouk K.O., Ahmed S.B.M., Elliott R.L., Benoit A., Alqahtani S.S., Ibrahim M.E., Bashir A.H.H., Alhoufie S.T.S., Elhassan G.O., Wales C.C. (2020). The Pentose phosphate pathway dynamics in cancer and its dependency on intracellular pH. Metabolites.

[8] Barber D.L., Liu Y. (2020). Intracellular pH regulates cancer and stem cell behaviors: A protein dynamics perspective. Front. Oncol..

[9] Harguindey S., Reshkin S.J. (2017). “The new pH-centric anticancer paradigm in Oncology and Medicine”; SCB, 2017. Semin. Cancer Biol..

[10] Harguindey S., Polo Orozco J., Alfarouk K.O., Devesa J. (2019). Hydrogen ion dynamics of cancer and a new molecular, biochemical and metabolic approach to the etiopathogenesis and treatment of brain malignancies. Int. J. Mol. Sci..

[11] Harguindey S., Alfarouk K., Orozco J.P., Hardonniere K., Stanciu D., Fais S., Devesa J. (2020). A new and integral approach to the etiopathogenesis and treatment of breast cancer based upon its hydrogen ion dynamics. Int. J. Mol. Sci..

[12] Warburg O., Posener K., Negelein E. (1924). Über den Stoffwechsel der Tumoren. Biochem. Z..

[13] Warburg O. (1925). The metabolism of carcinoma cells. J. Cancer Res..

[14] Warburg O. (1956). On the origin of cancer cells. Science.

[15] Warburg O. (1969). The prime cause and prevention of disease. Dr. Otto Warburg’s Address to Nobel Laureates, June 30, 1966 at Lindau, Lake Constance, Germany 1966.

[16] Burk D., Winzler R. (1944). The biochemistry of malignant tissue. Annu. Rev. Biochem..

[17] Warburg O., Christian W. (1936). Pyridin, der wasserstoffübertragende bestandteil von gärungsfermenten. Helv. Chim. Acta.

[18] Weinhouse S. (1956). On respiratory impairment in cancer cells. Science.

[19] DeBerardinis R.J., Chandel N.S. (2020). We need to talk about the Warburg effect. Nat. Metab..

[20] Halperin M.L., Connors H.P., Relman A.S., Karnovsky M.L. (1969). Factors that control the effect of pH on glycolysis in leukocytes. J. Biol. Chem..

[21] Relman A.S. (1972). Metabolic consequences of acid-base disorders. Kidney Int..

[22] Eagle H. (1974). Some effects of environmental pH on cellular metabolism and function. Control Prolif. Anim. Cells.

[23] Wilhelm G., Schulz J., Hofmann E. (1971). pH-dependence of aerobic glycolysis in Ehrlich ascites tumour cells. FEBS Lett..

[24] Rubin H., Fodge D. (1974). Interrelationships of glycolysis, sugar transport and the initiation of DNA synthesis in chick embryo cells. Control Prolif. Anim. Cells.

[25] Gevers W., Dowdle E. (1963). The effect of pH on glycolysis in vitro. Clin. Sci..

[26] Ui M. (1966). A role of phosphofructokinase in pH-dependent regulation of glycolysis. Biochim. Biophys. Acta.

[27] Lowenstein J.M., Chance B. (1968). The effect of hydrogen ions on the control of mitochondrial respiration. J. Biol. Chem..

[28] Alfarouk K.O., Verduzco D., Rauch C., Muddathir A.K., Adil H.H., Elhassan G.O., Ibrahim M.E., David Polo Orozco J., Cardone R.A., Reshkin S.J. (2014). Glycolysis, tumor metabolism, cancer growth and dissemination. A new pH-based etiopathogenic perspective and therapeutic approach to an old cancer question. Oncoscience.

[29] Warburg O.H. (1969). The prime cause and prevention of cancer. Lecture Delivered to Nobel Laureates on June 30, 1966 at Lindau, Lake Constance, Germany.

[30] Nagata H., Che X.F., Miyazawa K., Tomoda A., Konishi M., Ubukata H., Tabuchi T. (2011). Rapid decrease of intracellular pH associated with inhibition of Na^+^/H^+^ exchanger precedes apoptotic events in the *MNK45* and *MNK74* gastric cancer cell lines treated with 2-aminophenoxazine-3-one. Oncol. Rep..

[31] Xiao-Fang C., Zheng C.-L., Akiyama S.-I., Tomoda A. (2011). 2-Aminophenoxazine-3-one and 2-amino-4, 4α-dihydro-4α, 7-dimethyl-3H-phenoxazine-3-one cause cellular apoptosis by reducing higher intracellular pH in cancer cells. Proc. Jpn. Acad. Ser. B Phys. Biol. Sci..

[32] Quach C.H., Jung K.H., Lee J.H., Park J.W., Moon S.H., Cho Y.S., Choe Y.S., Lee K.H. (2016). Mild alkalization acutely triggers the Warburg effect by enhancing hexokinase activity via voltage-dependent anion channel binding. PLoS ONE.

[33] Harguindey S. (1994). Unitary strategy of cancerous cells: Hydrocarbon metabolism (1). Med. Interna.

[34] Harguindey S. (1994). The unitary strategy of cancer cells: The hydrogen ion as a multidimensional unitary factor and carbohydrate metabolism (2). An. Med. Interna (Madrid, Spain).

[35] Spugnini E.P., Sonveaux P., Stock C., Perez-Sayans M., De Milito A., Avnet S., Garcia A.G., Harguindey S., Fais S. (2015). Proton channels and exchangers in cancer. Biochim. Biophys. Acta.

[36] Reshkin S.J., Bellizzi A., Caldeira S., Albarani V., Malanchi I., Poignee M., Alunni-Fabbroni M., Casavola V., Tommasino M. (2000). Na^+^/H^+^ exchanger-dependent intracellular alkalinization is an early event in malignant transformation and plays an essential role in the development of subsequent transformation-associated phenotypes. FASEB J..

[37] Cardone R.A., Alfarouk K.O., Elliott R.L., Alqahtani S.S., Ahmed S.B.M., Aljarbou A.N., Greco M.R., Cannone S., Reshkin S.J. (2019). The role of sodium hydrogen exchanger 1 in dysregulation of proton dynamics and reprogramming of cancer metabolism as a sequela. Int. J. Mol. Sci..

[38] Amith S.R., Wilkinson J.M., Fliegel L. (2016). Na^+^/H^+^ exchanger NHE1 regulation modulates metastatic potential and epithelial-mesenchymal transition of triple-negative breast cancer cells. Oncotarget.

[39] Fliegel L. (2019). Structural and functional changes in the Na^+^/H^+^ exchanger isoform 1, induced by erk1/2 phosphorylation. Int. J. Mol. Sci..

[40] Alfarouk K.O., Ahmed S.B.M., Ahmed A., Elliott R.L., Ibrahim M.E., Ali H.S., Wales C.C., Nourwali I., Aljarbou A.N., Bashir A.H.H. (2020). The interplay of dysregulated pH and electrolyte imbalance in cancer. Cancers (Basel).

[41] Alfarouk K.O., Stock C.M., Taylor S., Walsh M., Muddathir A.K., Verduzco D., Bashir A.H., Mohammed O.Y., Elhassan G.O., Harguindey S. (2015). Resistance to cancer chemotherapy: Failure in drug response from ADME to P-gp. Cancer Cell Int..

[42] Alfarouk K.O., Muddathir A.K., Shayoub M.E. (2011). Tumor acidity as evolutionary spite. Cancers (Basel).

[43] Grillo-Hill B.K., Choi C., Jimenez-Vidal M., Barber D.L. (2015). Increased H^+^ efflux is sufficient to induce dysplasia and necessary for viability with oncogene expression. Elife.

[44] Amith S.R., Fliegel L. (2017). Na^+^/H^+^ exchanger-mediated hydrogen ion extrusion as a carcinogenic signal in triple-negative breast cancer etiopathogenesis and prospects for its inhibition in therapeutics. Semin. Cancer Biol..

[45] Amith S.R., Wilkinson J.M., Fliegel L. (2016). Assessing Na^+^/H^+^ exchange and cell effector functionality in metastatic breast cancer. Biochim. Open.

[46] Amith S.R., Fliegel L. (2013). Regulation of the Na^+^/H^+^ Exchanger (NHE1) in breast cancer metastasis. Cancer Res..

[47] Pethő Z., Najder K., Carvalho T., McMorrow R., Todesca L.M., Rugi M., Bulk E., Chan A., Löwik C.W.G.M., Reshkin S.J. (2020). pH-channeling in cancer: How pH-dependence of cation channels shapes cancer pathophysiology. Cancers (Basel).

[48] Lobo R.C., Hubbard N.E., Damonte P., Mori H., Penzvalto Z., Pham C., Koehne A.L., Go A.C., Anderson S.E., Cala P.M. (2016). glucose uptake and intracellular ph in a mouse model of ductal carcinoma in situ (DCIS) suggests metabolic heterogeneity. Front. Cell Dev. Biol..

[49] Ma Z., Yuan D., Cheng X., Tuo B., Liu X., Li T. (2020). Function of ion transporters in maintaining acid-base homeostasis of the mammary gland and the pathophysiological role in breast cancer. Am. J. Physiol. Regul. Integr. Comp. Physiol..

[50] Fliegel L. (2020). Role of pH regulatory proteins and dysregulation of pH in prostate cancer. Rev. Physiol. Biochem. Pharm..

[51] Amith S.R., Fong S., Baksh S., Fliegel L. (2015). Na ^+^/H ^+^exchange in the tumour microenvironment: Does NHE1 drive breast cancer carcinogenesis?. Int. J. Dev. Biol..

[52] Amith S.R., Wilkinson J.M., Baksh S., Fliegel L. (2015). The Na^+^/H^+^ exchanger (NHE1) as a novel co-adjuvant target in paclitaxel therapy of triple-negative breast cancer cells. Oncotarget.

[53] Zheng T., Jaattela M., Liu B. (2020). pH gradient reversal fuels cancer progression. Int. J. Biochem. Cell Biol..

[54] Lee S., Mele M., Vahl P., Christiansen P.M., Jensen V.E., Boedtkjer E. (2015). Na^+^, HCO_3_^−^ -cotransport is functionally upregulated during human breast carcinogenesis and required for the inverted pH gradient across the plasma membrane. Pflug. Arch..

[55] Andersen A.P., Samsoe-Petersen J., Oernbo E.K., Boedtkjer E., Moreira J.M.A., Kveiborg M., Pedersen S.F. (2018). The net acid extruders NHE1, NBCn1 and MCT4 promote mammary tumor growth through distinct but overlapping mechanisms. Int. J. Cancer.

[56] Flinck M., Kramer S.H., Schnipper J., Andersen A.P., Pedersen S.F. (2018). The acid-base transport proteins NHE1 and NBCn1 regulate cell cycle progression in human breast cancer cells. Cell Cycle.

[57] Cao L., Yuan Z., Liu M., Stock C. (2019). (Patho-)Physiology of Na^+^/H^+^ Exchangers (NHEs) in the digestive system. Front. Physiol..

[58] Li T., Tuo B. (2020). Pathophysiology of hepatic Na^+^/H^+^ exchange (Review). Exp. Med..

[59] Liu C.L., Zhang X., Liu J., Wang Y., Sukhova G.K., Wojtkiewicz G.R., Liu T., Tang R., Achilefu S., Nahrendorf M. (2019). Na^+^-H^+^ exchanger 1 determines atherosclerotic lesion acidification and promotes atherogenesis. Nat. Commun..

[60] Reshkin S.J., Cardone R.A., Harguindey S. (2013). Na^+^-H^+^ exchanger, pH regulation and cancer. Recent Pat. Anticancer Drug Discov..

[61] Boedtkjer E., Moreira J.M., Mele M., Vahl P., Wielenga V.T., Christiansen P.M., Jensen V.E., Pedersen S.F., Aalkjaer C. (2013). Contribution of Na^+^,HCO_3_^−^ -cotransport to cellular pH control in human breast cancer: A role for the breast cancer susceptibility locus *NBCn1* (*SLC4A7*). Int. J. Cancer.

[62] Lee S., Axelsen T.V., Andersen A.P., Vahl P., Pedersen S.F., Boedtkjer E. (2016). Disrupting Na^+^, HCO_3_^−^-cotransporter NBCn1 (Slc4a7) delays murine breast cancer development. Oncogene.

[63] Baenke F., Dubuis S., Brault C., Weigelt B., Dankworth B., Griffiths B., Jiang M., Mackay A., Saunders B., Spencer-Dene B. (2015). Functional screening identifies MCT4 as a key regulator of breast cancer cell metabolism and survival. J. Pathol..

[64] Loo S.Y., Chang M.K., Chua C.S., Kumar A.P., Pervaiz S., Clement M.V. (2012). NHE-1: A promising target for novel anti-cancer therapeutics. Curr. Pharm. Des..

[65] Pinheiro C., Sousa B., Albergaria A., Paredes J., Dufloth R., Vieira D., Schmitt F., Baltazar F. (2011). GLUT1 and CAIX expression profiles in breast cancer correlate with adverse prognostic factors and MCT1 overexpression. Histol. Histopathol..

[66] Hsieh M.J., Chen K.S., Chiou H.L., Hsieh Y.S. (2010). Carbonic anhydrase XII promotes invasion and migration ability of *MDA-MB-231* breast cancer cells through the p38 MAPK signaling pathway. Eur. J. Cell Biol..

[67] Neri D., Supuran C.T. (2011). Interfering with pH regulation in tumours as a therapeutic strategy. Nat. Rev. Drug Discov..

[68] Mboge M.Y., Mahon B.P., McKenna R., Frost S.C. (2018). Carbonic anhydrases: Role in pH control and cancer. Metabolites.

[69] Berrino E., Supuran C.T. (2019). Novel approaches for designing drugs that interfere with pH regulation. Expert Opin. Drug Discov..

[70] Lauritzen G., Stock C.M., Lemaire J., Lund S.F., Jensen M.F., Damsgaard B., Petersen K.S., Wiwel M., Ronnov-Jessen L., Schwab A. (2012). The Na^+^/H^+^ exchanger NHE1, but not the Na^+^, HCO_3_^−^ cotransporter NBCn1, regulates motility of *MCF7* breast cancer cells expressing constitutively active ErbB2. Cancer Lett..

[71] Chen C.H., Lee C.Z., Lin Y.C., Kao L.T., Lin H.C. (2019). Negative association of proton pump inhibitors with subsequent development of breast cancer: A nationwide population-based study. J. Clin. Pharm..

[72] Mihaila R.G. (2015). A minireview on NHE1 inhibitors. A rediscovered hope in oncohematology. Biomed. Pap. Med. Fac. Univ. Palacky Olomouc Czech. Repub..

[73] Goh W., Sleptsova-Freidrich I., Petrovic N. (2014). Use of proton pump inhibitors as adjunct treatment for triple-negative breast cancers. An introductory study. J. Pharm. Pharm. Sci..

[74] White K.A., Grillo-Hill B.K., Barber D.L. (2017). Cancer cell behaviors mediated by dysregulated pH dynamics at a glance. J. Cell Sci..

[75] Kakkad S., Krishnamachary B., Jacob D., Pacheco-Torres J., Goggins E., Bharti S.K., Penet M.F., Bhujwalla Z.M. (2019). Molecular and functional imaging insights into the role of hypoxia in cancer aggression. Cancer Metastasis Rev..

[76] Shimizu S., Eguchi Y., Kamiike W., Funahashi Y., Mignon A., Lacronique V., Matsuda H., Tsujimoto Y. (1998). Bcl-2 prevents apoptotic mitochondrial dysfunction by regulating proton flux. Proc. Natl. Acad. Sci. USA.

[77] Harguindey S., Pedraz J.L., Canero R.G., Katin M. (2000). Edelfosine, apoptosis, MDR and Na^+^/H^+^ exchanger: Induction mechanisms and treatment implications. Apoptosis.

[78] Lagadic-Gossmann D., Huc L., Lecureur V. (2004). Alterations of intracellular pH homeostasis in apoptosis: Origins and roles. Cell Death Differ..

[79] Bartosova M., Parkkila S., Pohlodek K., Karttunen T.J., Galbavy S., Mucha V., Harris A.L., Pastorek J., Pastorekova S. (2002). Expression of carbonic anhydrase IX in breast is associated with malignant tissues and is related to overexpression of c-erbB2. J. Pathol..

[80] Lou Y., McDonald P.C., Oloumi A., Chia S., Ostlund C., Ahmadi A., Kyle A., Auf dem Keller U., Leung S., Huntsman D. (2011). Targeting tumor hypoxia: Suppression of breast tumor growth and metastasis by novel carbonic anhydrase IX inhibitors. Cancer Res..

[81] Lock F.E., McDonald P.C., Lou Y., Serrano I., Chafe S.C., Ostlund C., Aparicio S., Winum J.Y., Supuran C.T., Dedhar S. (2013). Targeting carbonic anhydrase IX depletes breast cancer stem cells within the hypoxic niche. Oncogene.

[82] Meehan J., Ward C., Turnbull A., Bukowski-Wills J., Finch A.J., Jarman E.J., Xintaropoulou C., Martinez-Perez C., Gray M., Pearson M. (2017). Inhibition of pH regulation as a therapeutic strategy in hypoxic human breast cancer cells. Oncotarget.

[83] Yager J.D., Davidson N.E. (2006). Estrogen carcinogenesis in breast cancer. N. Engl. J. Med..

[84] Lloyd M.C., Alfarouk K.O., Verduzco D., Bui M.M., Gillies R.J., Ibrahim M.E., Brown J.S., Gatenby R.A. (2014). Vascular measurements correlate with estrogen receptor status. BMC Cancer.

[85] Gruvberger S., Ringner M., Chen Y., Panavally S., Saal L.H., Borg A., Ferno M., Peterson C., Meltzer P.S. (2001). Estrogen receptor status in breast cancer is associated with remarkably distinct gene expression patterns. Cancer Res..

[86] Morais-Santos F., Granja S., Miranda-Goncalves V., Moreira A.H., Queiros S., Vilaca J.L., Schmitt F.C., Longatto-Filho A., Paredes J., Baltazar F. (2015). Targeting lactate transport suppresses in vivo breast tumour growth. Oncotarget.

[87] Pinheiro C., Albergaria A., Paredes J., Sousa B., Dufloth R., Vieira D., Schmitt F., Baltazar F. (2010). Monocarboxylate transporter 1 is up-regulated in basal-like breast carcinoma. Histopathology.

[88] Payen V.L., Mina E., Van Hee V.F., Porporato P.E., Sonveaux P. (2019). Monocarboxylate transporters in cancer. Mol. Metab..

[89] Gorbatenko A., Olesen C.W., Boedtkjer E., Pedersen S.F. (2014). Regulation and roles of bicarbonate transporters in cancer. Front. Physiol..

[90] Boedtkjer E. (2019). Na^+^,HCO_3_^−^ cotransporter NBCn1 accelerates breast carcinogenesis. Cancer Metastasis Rev..

[91] Brisson L., Gillet L., Calaghan S., Besson P., Le Guennec J., Roger S., Gore J. (2011). NaV1. 5 enhances breast cancer cell invasiveness by increasing NHE1-dependent H+ efflux in caveolae. Oncogene.

[92] Fraser S.P., Diss J.K., Chioni A.M., Mycielska M.E., Pan H., Yamaci R.F., Pani F., Siwy Z., Krasowska M., Grzywna Z. (2005). Voltage-gated sodium channel expression and potentiation of human breast cancer metastasis. Clin. Cancer Res..

[93] Yang M., Kozminski D.J., Wold L.A., Modak R., Calhoun J.D., Isom L.L., Brackenbury W.J. (2012). Therapeutic potential for phenytoin: Targeting Na_v_1.5 sodium channels to reduce migration and invasion in metastatic breast cancer. Breast Cancer Res. Treat..

[94] O’Grady S., Morgan M.P. (2019). Calcium transport and signalling in breast cancer: Functional and prognostic significance. Seminars in Cancer Biology.

[95] Mitchell P. (1961). Coupling of phosphorylation to electron and hydrogen transfer by a chemi-osmotic type of mechanism. Nature.

[96] Cotter K., Liberman R., Sun-Wada G., Wada Y., Sgroi D., Naber S., Brown D., Breton S., Forgac M. (2016). The a3 isoform of subunit a of the vacuolar ATPase localizes to the plasma membrane of invasive breast tumor cells and is overexpressed in human breast cancer. Oncotarget.

[97] Daniel C., Bell C., Burton C., Harguindey S., Reshkin S.J., Rauch C. (2013). The role of proton dynamics in the development and maintenance of multidrug resistance in cancer. Biochim. Biophys. Acta.

[98] Von Schwarzenberg K., Lajtos T., Simon L., Muller R., Vereb G., Vollmar A.M. (2014). V-ATPase inhibition overcomes trastuzumab resistance in breast cancer. Mol. Oncol..

[99] Spugnini E.P., Citro G., Fais S. (2010). Proton pump inhibitors as anti vacuolar-ATPases drugs: A novel anticancer strategy. J. Exp. Clin. Cancer Res..

[100] Spugnini E.P., Fais S. (2020). Drug repurposing for anticancer therapies. A lesson from proton pump inhibitors. Expert Opin. Pat..

[101] Harguindey S., Arranz J.L., Wahl M.L., Orive G., Reshkin S.J. (2009). Proton transport inhibitors as potentially selective anticancer drugs. Anticancer Res..

[102] Brisson L., Driffort V., Benoist L., Poet M., Counillon L., Antelmi E., Rubino R., Besson P., Labbal F., Chevalier S. (2013). Na_v_1.5 Na+ channels allosterically regulate the NHE-1 exchanger and promote the activity of breast cancer cell invadopodia. J. Cell Sci..

[103] Bellizzi A., Greco M.R., Rubino R., Paradiso A., Forciniti S., Zeeberg K., Cardone R.A., Reshkin S.J. (2015). The scaffolding protein NHERF1 sensitizes EGFR-dependent tumor growth, motility and invadopodia function to gefitinib treatment in breast cancer cells. Int. J. Oncol..

[104] Schwab A., Stock C. (2014). Ion channels and transporters in tumour cell migration and invasion. Philos. Trans. R. Soc. Lond. B Biol. Sci..

[105] Nelson M., Yang M., Millican-Slater R., Brackenbury W.J. (2015). Nav1.5 regulates breast tumor growth and metastatic dissemination in vivo. Oncotarget.

[106] Gillet L., Roger S., Besson P., Lecaille F., Gore J., Bougnoux P., Lalmanach G., Le Guennec J.Y. (2009). Voltage-gated sodium channel activity promotes cysteine cathepsin-dependent invasiveness and colony growth of human cancer cells. J. Biol. Chem..

[107] Lu C., Ma Z., Cheng X., Wu H., Tuo B., Liu X., Li T. (2020). Pathological role of ion channels and transporters in the development and progression of triple-negative breast cancer. Cancer Cell Int..

[108] So C.L., Saunus J.M., Roberts-Thomson S.J., Monteith G.R. (2019). Calcium signalling and breast cancer. Semin. Cell Dev. Biol..

[109] Jonathan D., Josh H., Fukushiro-Lopes D.F., Laczynski D., Gentile S. (2017). Ion channels in breast cancer: From signaling to therapy. Breast Cancer Biol. Med..

[110] Yu C., Wang Y., Peng J., Shen Q., Chen M., Tang W., Li X., Cai C., Wang B., Cai S. (2017). Mitochondrial calcium uniporter as a target of microRNA-340 and promoter of metastasis via enhancing the Warburg effect. Oncotarget.

[111] Ward C., Meehan J., Gray M.E., Murray A.F., Argyle D.J., Kunkler I.H., Langdon S.P. (2020). The impact of tumour pH on cancer progression: Strategies for clinical intervention. Explor. Target. Anti Tumor Ther..

[112] Ibrahim-Hashim A., Estrella V. (2019). Acidosis and cancer: From mechanism to neutralization. Cancer Metastasis Rev..

[113] Montcourrier P., Silver I., Farnoud R., Bird I., Rochefort H. (1997). Breast cancer cells have a high capacity to acidify extracellular milieu by a dual mechanism. Clin. Exp. Metastasis.

[114] Huber V., Camisaschi C., Berzi A., Ferro S., Lugini L., Triulzi T., Tuccitto A., Tagliabue E., Castelli C., Rivoltini L. (2017). Cancer acidity: An ultimate frontier of tumor immune escape and a novel target of immunomodulation. Semin. Cancer Biol..

[115] Dumas J.F., Brisson L., Chevalier S., Maheo K., Fromont G., Moussata D., Besson P., Roger S. (2017). Metabolic reprogramming in cancer cells, consequences on pH and tumour progression: Integrated therapeutic perspectives with dietary lipids as adjuvant to anticancer treatment. Semin. Cancer Biol..

[116] Roma-Rodrigues C., Mendes R., Baptista P.V., Fernandes A.R. (2019). Targeting tumor microenvironment for cancer therapy. Int. J. Mol. Sci..

[117] Lacroix R., Rozeman E.A., Kreutz M., Renner K., Blank C.U. (2018). Targeting tumor-associated acidity in cancer immunotherapy. Cancer Immunol. Immunother..

[118] Pillai S.R., Damaghi M., Marunaka Y., Spugnini E.P., Fais S., Gillies R.J. (2019). Causes, consequences, and therapy of tumors acidosis. Cancer Metastasis Rev..

[119] Robey I.F., Baggett B.K., Kirkpatrick N.D., Roe D.J., Dosescu J., Sloane B.F., Hashim A.I., Morse D.L., Raghunand N., Gatenby R.A. (2009). Bicarbonate increases tumor pH and inhibits spontaneous metastases. Cancer Res..

[120] Fais S., Venturi G., Gatenby B. (2014). Microenvironmental acidosis in carcinogenesis and metastases: New strategies in prevention and therapy. Cancer Metastasis Rev..

[121] Pilon-Thomas S., Kodumudi K.N., El-Kenawi A.E., Russell S., Weber A.M., Luddy K., Damaghi M., Wojtkowiak J.W., Mule J.J., Ibrahim-Hashim A. (2016). Neutralization of tumor acidity improves antitumor responses to immunotherapy. Cancer Res..

[122] Calcinotto A., Filipazzi P., Grioni M., Iero M., De Milito A., Ricupito A., Cova A., Canese R., Jachetti E., Rossetti M. (2012). Modulation of microenvironment acidity reverses anergy in human and murine tumor-infiltrating T lymphocytes. Cancer Res..

[123] Wu H., Estrella V., Enriquez-Navas P., El-Kenawi A., Russell S., Abrahams D., Ibrahim-Hashim A., Longo D., Reshetnyak Y., Luddy K. (2019). Lymph nodes inhibit T-cell effector functions locally by establishing acidic niches. Biorxiv.

[124] Marches R., Vitetta E.S., Uhr J.W. (2001). A role for intracellular pH in membrane IgM-mediated cell death of human B lymphomas. Proc. Natl. Acad. Sci. USA.

[125] Thews O., Gassner B., Kelleher D.K., Schwerdt G., Gekle M. (2006). Impact of extracellular acidity on the activity of P-glycoprotein and the cytotoxicity of chemotherapeutic drugs. Neoplasia.

[126] Corbet C., Feron O. (2017). Tumour acidosis: From the passenger to the driver’s seat. Nat. Rev. Cancer.

[127] Thiebaut F., Currier S.J., Whitaker J., Haugland R.P., Gottesman M.M., Pastan I., Willingham M.C. (1990). Activity of the multidrug transporter results in alkalinization of the cytosol: Measurement of cytosolic pH by microinjection of a pH-sensitive dye. J. Histochem. Cytochem..

[128] Taylor S., Spugnini E.P., Assaraf Y.G., Azzarito T., Rauch C., Fais S. (2015). Microenvironment acidity as a major determinant of tumor chemoresistance: Proton pump inhibitors (PPIs) as a novel therapeutic approach. Drug Resist. Updat..

[129] Fais S. (2010). Proton pump inhibitor-induced tumour cell death by inhibition of a detoxification mechanism. J. Intern. Med..

[130] Weisburg J.H., Roepe P.D., Dzekunov S., Scheinberg D.A. (1999). Intracellular pH and multidrug resistance regulate complement-mediated cytotoxicity of nucleated human cells. J. Biol. Chem..

[131] Alfarouk K.O. (2016). Tumor metabolism, cancer cell transporters, and microenvironmental resistance. J. Enzym. Inhib. Med. Chem..

[132] Thews O., Nowak M., Sauvant C., Gekle M. (2011). Hypoxia-induced extracellular acidosis increases p-glycoprotein activity and chemoresistance in tumors in vivo via p38 signaling pathway. Oxygen Transport to Tissue XXXII.

[133] Damaghi M., Gillies R. (2017). Phenotypic changes of acid-adapted cancer cells push them toward aggressiveness in their evolution in the tumor microenvironment. Cell Cycle.

[134] Gupta S.C., Singh R., Pochampally R., Watabe K., Mo Y.Y. (2014). Acidosis promotes invasiveness of breast cancer cells through ROS-AKT-NF-kappaB pathway. Oncotarget.

[135] Di Pompo G., Lemma S., Canti L., Rucci N., Ponzetti M., Errani C., Donati D.M., Russell S., Gillies R., Chano T. (2017). Intratumoral acidosis fosters cancer-induced bone pain through the activation of the mesenchymal tumor-associated stroma in bone metastasis from breast carcinoma. Oncotarget.

[136] Rothberg J.M., Bailey K.M., Wojtkowiak J.W., Ben-Nun Y., Bogyo M., Weber E., Moin K., Blum G., Mattingly R.R., Gillies R.J. (2013). Acid-mediated tumor proteolysis: Contribution of cysteine cathepsins. Neoplasia.

[137] Chen Z., Ai L., Mboge M.Y., Tu C., McKenna R., Brown K.D., Heldermon C.D., Frost S.C. (2018). Differential expression and function of CAIX and CAXII in breast cancer: A comparison between tumorgraft models and cells. PLoS ONE.

[138] Mouridsen H.T. (2007). Letrozole in advanced breast cancer: The PO25 trial. Breast Cancer Res. Treat..

[139] Reshkin S.J., Bellizzi A., Cardone R.A., Tommasino M., Casavola V., Paradiso A. (2003). Paclitaxel induces apoptosis via protein kinase A- and p38 mitogen-activated protein-dependent inhibition of the Na^+^/H^+^ exchanger (NHE) NHE isoform 1 in human breast cancer cells. Clin. Cancer Res..

[140] Supuran C.T. (2018). Carbonic anhydrases and metabolism. Metabolites.

[141] Lim B., Woodward W.A., Wang X., Reuben J.M., Ueno N.T. (2018). Inflammatory breast cancer biology: The tumour microenvironment is key. Nat. Rev. Cancer.

[142] Kaloyianni M., Bourikas D., Koliakos G. (2001). The effect of insulin on Na^+^-H^+^ antiport activity of obese and normal subjects erythrocytes. Cell. Physiol. Biochem..

[143] Moore R.D., Gupta R.K. (1980). Effect of insulin on intracellular pH as observed by 31P NMR spectroscopy. Int. J. Quantum Chem..

[144] Williams B., Howard R.L. (1994). Glucose-induced changes in Na^+^/H^+^ antiport activity and gene expression in cultured vascular smooth muscle cells. Role of protein kinase C. J. Clin. Investig..

[145] Wani B., Aziz S.A., Ganaie M.A., Mir M.H. (2017). Metabolic syndrome and breast cancer risk. Indian J. Med. Paediatr. Oncol..

[146] Tsujimoto T., Kajio H., Sugiyama T. (2017). Association between hyperinsulinemia and increased risk of cancer death in nonobese and obese people: A population-based observational study. Int. J. Cancer.

[147] Ramirez M.A., Beltran A.R., Araya J.E., Cornejo M., Toledo F., Fuentes G., Sobrevia L. (2019). Involvement of intracellular pH in vascular insulin resistance. Curr. Vasc. Pharm..

[148] Tuccori M., Wu J.W., Yin H., Majdan A., Azoulay L. (2015). The use of glyburide compared with other sulfonylureas and the risk of cancer in patients with type 2 diabetes. Diabetes Care.

[149] Pasello G., Urso L., Conte P., Favaretto A. (2013). Effects of sulfonylureas on tumor growth: A review of the literature. Oncologist.

[150] Gao R., Yang T., Xu W. (2017). Enemies or weapons in hands: Investigational anti-diabetic drug glibenclamide and cancer risk. Expert Opin. Investig. Drugs.

[151] Boyd D.B. (2003). Insulin and cancer. Integr. Cancer.

[152] Arcidiacono B., Iiritano S., Nocera A., Possidente K., Nevolo M.T., Ventura V., Foti D., Chiefari E., Brunetti A. (2012). Insulin resistance and cancer risk: An overview of the pathogenetic mechanisms. Exp. Diabetes Res..

[153] Ruiz-Narvaez E.A., Lunetta K.L., Hong C.C., Haddad S., Yao S., Cheng T.D., Bensen J.T., Bandera E.V., Haiman C.A., Troester M.A. (2016). Genetic variation in the insulin, insulin-like growth factor, growth hormone, and leptin pathways in relation to breast cancer in African-American women: The AMBER consortium. NPJ Breast Cancer.

[154] Koedoot E., Fokkelman M., Rogkoti V.M., Smid M., van de Sandt I., de Bont H., Pont C., Klip J.E., Wink S., Timmermans M.A. (2019). Uncovering the signaling landscape controlling breast cancer cell migration identifies novel metastasis driver genes. Nat. Commun..

[155] Harguindey S., Orive G., Luis Pedraz J., Paradiso A., Reshkin S.J. (2005). The role of pH dynamics and the Na^+^/H^+^ antiporter in the etiopathogenesis and treatment of cancer. Two faces of the same coin—One single nature. Biochim. Biophys. Acta.

[156] Evans J.L., Maddux B.A., Goldfine I.D. (2005). The molecular basis for oxidative stress-induced insulin resistance. Antioxid. Redox Signal..

[157] Wu Y., Gao B., Xiong Q.J., Wang Y.C., Huang D.K., Wu W.N. (2017). Acid-sensing ion channels contribute to the effect of extracellular acidosis on proliferation and migration of *A549* cells. Tumour Biol..

[158] Fine E.J., Segal-Isaacson C.J., Feinman R.D., Herszkopf S., Romano M.C., Tomuta N., Bontempo A.F., Negassa A., Sparano J.A. (2012). Targeting insulin inhibition as a metabolic therapy in advanced cancer: A pilot safety and feasibility dietary trial in 10 patients. Nutrition.

[159] Lann D., LeRoith D. (2008). The role of endocrine insulin-like growth factor-I and insulin in breast cancer. J. Mammary Gland Biol. Neoplasia.

[160] Gunter M.J., Hoover D.R., Yu H., Wassertheil-Smoller S., Rohan T.E., Manson J.E., Li J., Ho G.Y., Xue X., Anderson G.L. (2009). Insulin, insulin-like growth factor-I, and risk of breast cancer in postmenopausal women. J. Natl. Cancer Inst..

[161] Clevenger C.V., Furth P.A., Hankinson S.E., Schuler L.A. (2003). The role of prolactin in mammary carcinoma. Endocr. Rev..

[162] Pedraz-Cuesta E., Fredsted J., Jensen H.H., Bornebusch A., Nejsum L.N., Kragelund B.B., Pedersen S.F. (2016). Prolactin signaling stimulates invasion via Na^+^/H^+^ Exchanger NHE1 in *T47D* human breast cancer cells. Mol. Endocrinol..

[163] MohanKumar S.M., Kasturi B.S., Shin A.C., Balasubramanian P., Gilbreath E.T., Subramanian M., Mohankumar P.S. (2011). Chronic estradiol exposure induces oxidative stress in the hypothalamus to decrease hypothalamic dopamine and cause hyperprolactinemia. Am. J. Physiol. Regul. Integr. Comp. Physiol..

[164] Wennbo H., Gebre-Medhin M., Gritli-Linde A., Ohlsson C., Isaksson O.G., Tornell J. (1997). Activation of the prolactin receptor but not the growth hormone receptor is important for induction of mammary tumors in transgenic mice. J. Clin. Investig..

[165] Rich I.N., Worthington-White D., Garden O.A., Musk P. (2000). Apoptosis of leukemic cells accompanies reduction in intracellular pH after targeted inhibition of the Na^+^/H^+^ exchanger. Blood.

[166] Hawsawi Y.M., Al-Numair N.S., Sobahy T.M., Al-Ajmi A.M., Al-Harbi R.M., Baghdadi M.A., Oyouni A.A., Alamer O.M. (2019). The role of *BRCA1/2* in hereditary and familial breast and ovarian cancers. Mol. Genet. Genom. Med..

[167] Tavares-Valente D., Baltazar F., Moreira R., Queiros O. (2013). Cancer cell bioenergetics and pH regulation influence breast cancer cell resistance to paclitaxel and doxorubicin. J. Bioenerg. Biomembr..

[168] Rauch C., Blanchard A., Wood E., Dilion E., Wahl M.L., Harguindey S., Meszaros A., Balogh G. (2009). Cell membranes, cytosolic pH and drug transport in cancer and MDR: Physics, biochemistry and molecular biology. Multiple Drug Resistance.

[169] Murakami T., Shibuya I., Ise T., Chen Z.S., Akiyama S., Nakagawa M., Izumi H., Nakamura T., Matsuo K., Yamada Y. (2001). Elevated expression of vacuolar proton pump genes and cellular pH in cisplatin resistance. Int. J. Cancer.

[170] Rauch C. (2009). On the relationship between drug’s size, cell membrane mechanical properties and high levels of multi drug resistance: A comparison to published data. Eur. Biophys. J..

[171] Omran Z., Scaife P., Stewart S., Rauch C. (2017). Physical and biological characteristics of multi drug resistance (MDR): An integral approach considering pH and drug resistance in cancer. Semin. Cancer Biol..

[172] Keizer H.G., Joenje H. (1989). Increased cytosolic pH in multidrug-resistant human lung tumor cells: Effect of verapamil. J. Natl. Cancer Inst..

[173] Wei L.Y., Roepe P.D. (1994). Low external pH and osmotic shock increase the expression of human MDR protein. Biochemistry.

[174] Roepe P.D. (2001). pH and multidrug resistance. Novartis Foundation Symposia.

[175] Chen Q., Liu Y., Zhu X.L., Feng F., Yang H., Xu W. (2019). Increased NHE1 expression is targeted by specific inhibitor cariporide to sensitize resistant breast cancer cells to doxorubicin in vitro and in vivo. BMC Cancer.

[176] Rath S., Liebl J., Furst R., Vollmar A.M., Zahler S. (2014). Regulation of endothelial signaling and migration by v-ATPase. Angiogenesis.

[177] Boscoboinik D., Gupta R.S., Epand R.M. (1990). Investigation of the relationship between altered intracellular pH and multidrug resistance in mammalian cells. Br. J. Cancer.

[178] Epand R.F., Epand R.M., Gupta R.S., Cragoe E.J. (1991). Reversal of intrinsic multidrug resistance in Chinese hamster ovary cells by amiloride analogs. Br. J. Cancer.

[179] Roepe P.D., Wei L.Y., Cruz J., Carlson D. (1993). Lower electrical membrane potential and altered pHi homeostasis in multidrug-resistant (MDR) cells: Further characterization of a series of MDR cell lines expressing different levels of P-glycoprotein. Biochemistry.

[180] Porporato P.E., Dhup S., Dadhich R.K., Copetti T., Sonveaux P. (2011). Anticancer targets in the glycolytic metabolism of tumors: A comprehensive review. Front. Pharm..

[181] Barriere H., Poujeol C., Tauc M., Blasi J.M., Counillon L., Poujeol P. (2001). CFTR modulates programmed cell death by decreasing intracellular pH in Chinese hamster lung fibroblasts. Am. J. Physiol. Cell Physiol..

[182] Harguindey S., Gonzalez Molinillo J., Chinchilla D., Reshkin S., Tomoda A. (2011). Further along a clinical protocol using a cocktail of PTIs in human cancer. ISPDC Abstract Book. 2nd ISPD Meeting, Nice, France, 18–19 November, 2011.

[183] Horvat B., Taheri S., Salihagic A. (1992). Tumour cell proliferation is abolished by inhibitors of Na^+^/H^+^ and HCO_3_- Cl- exchange. Eur. J. Cancer.

[184] Cone C.D. (1971). Unified theory on the basic mechanism of normal mitotic control and oncogenesis. J. Biol..

[185] Sparks R.L., Pool T.B., Smith N.K., Cameron I.L. (1983). Effects of amiloride on tumor growth and intracellular element content of tumor cells in vivo. Cancer Res..

[186] Roger S., Besson P., Le Guennec J.Y. (2003). Involvement of a novel fast inward sodium current in the invasion capacity of a breast cancer cell line. Biochim. Biophys. Acta.

[187] Pouyssegur J., Chambard J.C., Franchi A., Paris S., Van Obberghen-Schilling E. (1982). Growth factor activation of an amiloride-sensitive Na^+^/H^+^ exchange system in quiescent fibroblasts: Coupling to ribosomal protein S6 phosphorylation. Proc. Natl. Acad. Sci. USA.

[188] He B., Zhang M., Zhu R. (2010). Na^+^/H^+^ exchanger blockade inhibits the expression of vascular endothelial growth factor in *SGC7901* cells. Oncol. Rep..

[189] Kellen J.A., Mirakian A., Kolin A. (1988). Antimetastatic effect of amiloride in an animal tumour model. Anticancer Res..

[190] Evans D.M., Sloan-Stakleff K., Arvan M., Guyton D.P. (1998). Time and dose dependency of the suppression of pulmonary metastases of rat mammary cancer by amiloride. Clin. Exp. Metastasis.

[191] Matthews H., Ranson M., Kelso M.J. (2011). Anti-tumour/metastasis effects of the potassium-sparing diuretic amiloride: An orally active anti-cancer drug waiting for its call-of-duty?. Int. J. Cancer.

[192] Harguindey S., Cragoe E.J., Kleyman T.R., Simchowitz L. (1992). Use of Na^+^/H^+^ antiporter inhibitors as a novel approach to cancer treatment. Amiloride and Its Analogs: Unique Cation Transport Inhibitors.

[193] Alliegro M.C., Alliegro M.A., Cragoe E.J., Glaser B.M. (1993). Amiloride inhibition of angiogenesis in vitro. J. Exp. Zool..

[194] He B., Deng C., Zhang M., Zou D., Xu M. (2007). Reduction of intracellular pH inhibits the expression of VEGF in *K562* cells after targeted inhibition of the Na^+^/H^+^ exchanger. Leuk. Res..

[195] Harguindey S., Pedraz J.L., Garcia Canero R., Perez de Diego J., Cragoe E.J. (1995). Hydrogen ion-dependent oncogenesis and parallel new avenues to cancer prevention and treatment using a H^+^-mediated unifying approach: pH-related and pH-unrelated mechanisms. Crit. Rev. Oncog..

[196] Harguindey S., Orive G., Pedraz J.L., Bello G., Arranz J.L., Samaniego J.M. (2002). Apparent cure of a case of metastatic ovarian carcinoma after the chronic treatment with Na^+^H^+^ antiport inhibitors. Oncologia.

[197] Orive G., Reshkin S.J., Harguindey S., Pedraz J.L. (2003). Hydrogen ion dynamics and the Na^+^/H^+^ exchanger in cancer angiogenesis and antiangiogenesis. Br. J. Cancer.

[198] Nocentini A., Supuran C.T. (2018). Carbonic anhydrase inhibitors as antitumor/antimetastatic agents: A patent review (2008–2018). Expert Opin. Pat..

[199] Federici C., Lugini L., Marino M.L., Carta F., Iessi E., Azzarito T., Supuran C.T., Fais S. (2016). Lansoprazole and carbonic anhydrase IX inhibitors sinergize against human melanoma cells. J. Enzym. Inhib. Med. Chem..

[200] Supuran C.T. (2017). Carbonic anhydrase inhibition and the management of hypoxic tumors. Metabolites.

[201] Andreucci E., Ruzzolini J., Peppicelli S., Bianchini F., Laurenzana A., Carta F., Supuran C.T., Calorini L. (2019). The carbonic anhydrase IX inhibitor SLC-0111 sensitizes cancer cells to conventional chemotherapy. J. Enzym. Inhib. Med. Chem..

[202] Gieling R.G., Parker C.A., De Costa L.A., Robertson N., Harris A.L., Stratford I.J., Williams K.J. (2013). Inhibition of carbonic anhydrase activity modifies the toxicity of doxorubicin and melphalan in tumour cells in vitro. J. Enzym. Inhib. Med. Chem..

[203] Mboge M.Y., Chen Z., Wolff A., Mathias J.V., Tu C., Brown K.D., Bozdag M., Carta F., Supuran C.T., McKenna R. (2018). Selective inhibition of carbonic anhydrase IX over carbonic anhydrase XII in breast cancer cells using benzene sulfonamides: Disconnect between activity and growth inhibition. PLoS ONE.

[204] Marathe K., McVicar N., Li A., Bellyou M., Meakin S., Bartha R. (2016). Topiramate induces acute intracellular acidification in glioblastoma. J. Neurooncol..

[205] Albatany M., Meakin S., Bartha R. (2018). The Monocarboxylate transporter inhibitor Quercetin induces intracellular acidification in a mouse model of Glioblastoma Multiforme: In-vivo detection using magnetic resonance imaging. Investig. New Drugs.

[206] Srivastava S., Somasagara R.R., Hegde M., Nishana M., Tadi S.K., Srivastava M., Choudhary B., Raghavan S.C. (2016). Quercetin, a natural flavonoid interacts with DNA, arrests cell cycle and causes tumor regression by activating mitochondrial pathway of apoptosis. Sci. Rep..

[207] Marchiq I., Pouyssegur J. (2016). Hypoxia, cancer metabolism and the therapeutic benefit of targeting lactate/H^+^ symporters. J. Mol. Med. (Berl.).

[208] Granja S., Tavares-Valente D., Queiros O., Baltazar F. (2017). Value of pH regulators in the diagnosis, prognosis and treatment of cancer. Semin. Cancer Biol..

[209] Parks S.K., Cormerais Y., Pouyssegur J. (2017). Hypoxia and cellular metabolism in tumour pathophysiology. J. Physiol..

[210] Long Y., Gao Z., Hu X., Xiang F., Wu Z., Zhang J., Han X., Yin L., Qin J., Lan L. (2018). Downregulation of *MCT 4* for lactate exchange promotes the cytotoxicity of NK cells in breast carcinoma. Cancer Med..

[211] Nath K., Guo L., Nancolas B., Nelson D.S., Shestov A.A., Lee S.C., Roman J., Zhou R., Leeper D.B., Halestrap A.P. (2016). Mechanism of antineoplastic activity of lonidamine. Biochim. Biophys. Acta.

[212] McIntyre A., Hulikova A., Ledaki I., Snell C., Singleton D., Steers G., Seden P., Jones D., Bridges E., Wigfield S. (2016). Disrupting hypoxia-induced bicarbonate transport acidifies tumor cells and suppresses tumor growth. Cancer Res..

[213] Rotin D., Wan P., Grinstein S., Tannock I. (1987). Cytotoxicity of compounds that interfere with the regulation of intracellular pH: A potential new class of anticancer drugs. Cancer Res..

[214] Tannock I.F., Rotin D. (1989). Acid pH in tumors and its potential for therapeutic exploitation. Cancer Res..

[215] Mokhtari R.B., Baluch N., Tsui M.K.H., Kumar S., Homayouni T.S., Aitken K., Das B., Baruchel S., Yeger H. (2017). Acetazolamide potentiates the anti-tumor potential of HDACi, MS-275, in neuroblastoma. BMC Cancer.

[216] Cazzamalli S., Corso A.D., Neri D. (2017). Linker stability influences the anti-tumor activity of acetazolamide-drug conjugates for the therapy of renal cell carcinoma. J. Control. Release.

[217] Gao H., Dong H., Li G., Jin H. (2018). Combined treatment with acetazolamide and cisplatin enhances chemosensitivity in laryngeal carcinoma *Hep-2* cells. Oncol. Lett..

[218] Wu L., Bernal G.M., Cahill K.E., Pytel P., Fitzpatrick C.A., Mashek H., Weichselbaum R.R., Yamini B. (2018). *BCL3* expression promotes resistance to alkylating chemotherapy in gliomas. Sci. Transl. Med..

[219] Perez-Sayans M., Somoza-Martin J.M., Barros-Angueira F., Rey J.M., Garcia-Garcia A. (2009). V-ATPase inhibitors and implication in cancer treatment. Cancer Treat. Rev..

[220] Zhang H., Lu J., Liu J., Zhang G., Lu A. (2020). Advances in the discovery of exosome inhibitors in cancer. J. Enzym. Inhib. Med. Chem..

[221] Spugnini E.P., Buglioni S., Carocci F., Francesco M., Vincenzi B., Fanciulli M., Fais S. (2014). High dose lansoprazole combined with metronomic chemotherapy: A phase I/II study in companion animals with spontaneously occurring tumors. J. Transl. Med..

[222] Lu Z.N., Tian B., Guo X.L. (2017). Repositioning of proton pump inhibitors in cancer therapy. Cancer Chemother. Pharm..

[223] Wang B.Y., Zhang J., Wang J.L., Sun S., Wang Z.H., Wang L.P., Zhang Q.L., Lv F.F., Cao E.Y., Shao Z.M. (2015). Intermittent high dose proton pump inhibitor enhances the antitumor effects of chemotherapy in metastatic breast cancer. J. Exp. Clin. Cancer Res..

[224] Wang X., Liu C., Wang J., Fan Y., Wang Z., Wang Y. (2017). Proton pump inhibitors increase the chemosensitivity of patients with advanced colorectal cancer. Oncotarget.

[225] Ding D.C., Sung F.C., Chen W., Wang J.H., Lin S.Z. (2019). Proton pump inhibitors reduce breast cancer risk in gastric ulcer patients: A population-based cohort study. Breast J..

[226] Harguindey S., DeCastro L., Barcos M., Getaz E.P., Henderson E.S., Freeman A. (1979). Hypercalcemia complicating childhood malignancies: A report of seven cases with some pathophysiological considerations. Cancer.

[227] Avnet S., Lemma S., Cortini M., Pellegrini P., Perut F., Zini N., Kusuzaki K., Chano T., Grisendi G., Dominici M. (2016). Altered pH gradient at the plasma membrane of osteosarcoma cells is a key mechanism of drug resistance. Oncotarget.

[228] Hiasa M., Okui T., Allette Y.M., Ripsch M.S., Sun-Wada G.H., Wakabayashi H., Roodman G.D., White F.A., Yoneda T. (2017). Bone pain induced by multiple myeloma is reduced by targeting V-ATPase and ASIC3. Cancer Res..

[229] Deval E., Noel J., Lay N., Alloui A., Diochot S., Friend V., Jodar M., Lazdunski M., Lingueglia E. (2008). ASIC3, a sensor of acidic and primary inflammatory pain. EMBO J..

[230] Hoang B.X., Shaw D.G., Han B., Fang J.Y., Nimni M. (2015). Acidosis and formaldehyde secretion as a possible pathway of cancer pain and options for improved cancer pain control. J. Pain Palliat. Care Pharm..

[231] Hoang B.X., Tran H.Q., Vu U.V., Pham Q.T., Shaw D.G. (2014). Palliative treatment for advanced biliary adenocarcinomas with combination dimethyl sulfoxide-sodium bicarbonate infusion and S-adenosyl-L-methionine. J. Pain Palliat. Care Pharm..

[232] Hoang B.X., Tran D.M., Tran H.Q., Nguyen P.T., Pham T.D., Dang H.V., Ha T.V., Tran H.D., Hoang C., Luong K.N. (2011). Dimethyl sulfoxide and sodium bicarbonate in the treatment of refractory cancer pain. J. Pain Palliat. Care Pharm..

[233] Ibrahim-Hashim A., Wojtkowiak J.W., de Lourdes Coelho Ribeiro M., Estrella V., Bailey K.M., Cornnell H.H., Gatenby R.A., Gillies R.J. (2011). Free base lysine increases survival and reduces metastasis in prostate cancer model. J. Cancer Sci..

[234] Xu R., Ji Z., Xu C., Zhu J. (2018). The clinical value of using chloroquine or hydroxychloroquine as autophagy inhibitors in the treatment of cancers: A systematic review and meta-analysis. Medicine.

[235] Tvingsholm S.A., Dehlendorff C., Østerlind K., Friis S., Jäättelä M. (2018). Proton pump inhibitor use and cancer mortality. Int. J. Cancer.

[236] Fairhurst C., Watt I., Martin F., Bland M., Brackenbury W.J. (2014). Exposure to sodium channel-inhibiting drugs and cancer survival: Protocol for a cohort study using the QResearch primary care database. BMJ Open.

[237] Fraser S.P., Pardo L.A. (2008). Ion channels: Functional expression and therapeutic potential in cancer. colloquium on ion channels and cancer. EMBO Rep..

[238] Fairhurst C., Watt I., Martin F., Bland M., Brackenbury W.J. (2015). Sodium channel-inhibiting drugs and survival of breast, colon and prostate cancer: A population-based study. Sci. Rep..

[239] Sohn J.H., Kim Y.T., Rha S.Y., Yoo N.C., Roh J.K., Kim B.S., Suh C.O., Kim G.E., Jang W.I., Chung H.C. (2003). Paclitaxel and cisplatin combination chemotherapy in pretreated breast cancer. Cancer Res. Treat..

[240] Turner N.C., Tutt A.N. (2012). Platinum chemotherapy for *BRCA1*-related breast cancer: Do we need more evidence?. Breast Cancer Res..

[241] Elserafi M.M., Zeeneldin A.A., Abdelsalam I.M., Nassar H.R., Moneer M.M., Buhoush W.H. (2018). First-line paclitaxel and cisplatin used sequentially or in combination in metastatic breast cancer: A phase II randomized study. J. Egypt. Natl. Canc. Inst..

[242] Rosenberg B. (1980). Cisplatin: Its history and possible mechanisms of action. Cisplatin.

[243] Dasari S., Tchounwou P.B. (2014). Cisplatin in cancer therapy: Molecular mechanisms of action. Eur. J. Pharm..

[244] Raudenska M., Balvan J., Fojtu M., Gumulec J., Masarik M. (2019). Unexpected therapeutic effects of cisplatin. Metallomics.

[245] Makovec T. (2019). Cisplatin and beyond: Molecular mechanisms of action and drug resistance development in cancer chemotherapy. Radiol. Oncol..

[246] Shirmanova M.V., Druzhkova I.N., Lukina M.M., Dudenkova V.V., Ignatova N.I., Snopova L.B., Shcheslavskiy V.I., Belousov V.V., Zagaynova E.V. (2017). Chemotherapy with cisplatin: Insights into intracellular pH and metabolic landscape of cancer cells in vitro and in vivo. Sci. Rep..

[247] Shetti D., Zhang B., Fan C., Mo C., Lee B.H., Wei K. (2019). Low dose of paclitaxel combined with XAV939 attenuates metastasis, angiogenesis and growth in breast cancer by suppressing wnt signaling. Cells.

[248] Cardone R.A., Greco M.R., Zeeberg K., Zaccagnino A., Saccomano M., Bellizzi A., Bruns P., Menga M., Pilarsky C., Schwab A. (2015). A novel NHE1-centered signaling cassette drives epidermal growth factor receptor-dependent pancreatic tumor metastasis and is a target for combination therapy. Neoplasia.

[249] Yan L., Shen J., Wang J., Yang X., Dong S., Lu S. (2020). Nanoparticle-based drug delivery system: A patient-friendly chemotherapy for oncology. Dose Response.

[250] Samavat H., Kurzer M.S. (2015). Estrogen metabolism and breast cancer. Cancer Lett..

[251] Dauchy R.T., Xiang S., Mao L., Brimer S., Wren M.A., Yuan L., Anbalagan M., Hauch A., Frasch T., Rowan B.G. (2014). Circadian and melatonin disruption by exposure to light at night drives intrinsic resistance to tamoxifen therapy in breast cancer. Cancer Res..

[252] Lega I.C., Austin P.C., Gruneir A., Goodwin P.J., Rochon P.A., Lipscombe L.L. (2013). Association between metformin therapy and mortality after breast cancer: A population-based study. Diabetes Care.

[253] Roshan M.H., Shing Y.K., Pace N.P. (2019). Metformin as an adjuvant in breast cancer treatment. SAGE Open Med..

[254] Grover-McKay M., Walsh S.A., Seftor E.A., Thomas P.A., Hendrix M.J. (1998). Role for glucose transporter 1 protein in human breast cancer. Pathol. Oncol. Res..

[255] Garrido P., Osorio F.G., Moran J., Cabello E., Alonso A., Freije J.M., Gonzalez C. (2015). Loss of GLUT4 induces metabolic reprogramming and impairs viability of breast cancer cells. J. Cell. Physiol..

[256] Wang J., Li G., Wang Y., Tang S., Sun X., Feng X., Li Y., Bao G., Li P., Mao X. (2015). Suppression of tumor angiogenesis by metformin treatment via a mechanism linked to targeting of HER2/HIF-1α/VEGF secretion axis. Oncotarget.

[257] Schexnayder C., Broussard K., Onuaguluchi D., Poche A., Ismail M., McAtee L., Llopis S., Keizerweerd A., McFerrin H., Williams C. (2018). Metformin inhibits migration and invasion by suppressing ROS production and COX2 expression in *MDA-MB-231* breast cancer Cells. Int. J. Mol. Sci..

[258] Fan C., Wang Y., Liu Z., Sun Y., Wang X., Wei G., Wei J. (2015). Metformin exerts anticancer effects through the inhibition of the Sonic hedgehog signaling pathway in breast cancer. Int. J. Mol. Med..

[259] Riobo-Del Galdo N.A., Lara Montero A., Wertheimer E.V. (2019). Role of hedgehog signaling in breast cancer: Pathogenesis and therapeutics. Cells.

[260] Iliopoulos D., Hirsch H.A., Struhl K. (2011). Metformin decreases the dose of chemotherapy for prolonging tumor remission in mouse xenografts involving multiple cancer cell types. Cancer Res..

[261] Vazquez-Martin A., Oliveras-Ferraros C., Del Barco S., Martin-Castillo B., Menendez J.A. (2011). The anti-diabetic drug metformin suppresses self-renewal and proliferation of trastuzumab-resistant tumor-initiating breast cancer stem cells. Breast Cancer Res. Treat..

[262] Leone A., Di Gennaro E., Bruzzese F., Avallone A., Budillon A. (2014). New perspective for an old antidiabetic drug: Metformin as anticancer agent. Cancer Treat. Res..

[263] De A., Kuppusamy G. (2019). Metformin in breast cancer: Preclinical and clinical evidence. Curr. Probl. Cancer.

[264] Bayraktar S., Hernadez-Aya L.F., Lei X., Meric-Bernstam F., Litton J.K., Hsu L., Hortobagyi G.N., Gonzalez-Angulo A.M. (2012). Effect of metformin on survival outcomes in diabetic patients with triple receptor-negative breast cancer. Cancer.

[265] Qu H., Yang X. (2015). Metformin inhibits angiogenesis induced by interaction of hepatocellular carcinoma with hepatic stellate cells. Cell Biochem. Biophys..

[266] Guimaraes T.A., Farias L.C., Santos E.S., de Carvalho Fraga C.A., Orsini L.A., de Freitas Teles L., Feltenberger J.D., de Jesus S.F., de Souza M.G., Santos S.H. (2016). Metformin increases PDH and suppresses HIF-1alpha under hypoxic conditions and induces cell death in oral squamous cell carcinoma. Oncotarget.

[267] Han J., Li Y., Liu X., Zhou T., Sun H., Edwards P., Gao H., Yu F.S., Qiao X. (2018). Metformin suppresses retinal angiogenesis and inflammation in vitro and in vivo. PLoS ONE.

[268] Ni H.Z., Liu Z., Sun L.L., Zhou M., Liu C., Li W.D., Li X.Q. (2019). Metformin inhibits angiogenesis of endothelial progenitor cells via miR-221-mediated p27 expression and autophagy. Future Med. Chem..

[269] Blagosklonny M.V. (2001). Hypoxia-inducible factor: Achilles’ heel of antiangiogenic cancer therapy (review). Int. J. Oncol..

[270] De Francesco E.M., Lappano R., Santolla M.F., Marsico S., Caruso A., Maggiolini M. (2013). HIF-1alpha/GPER signaling mediates the expression of VEGF induced by hypoxia in breast cancer associated fibroblasts (CAFs). Breast Cancer Res..

[271] Briggs K.J., Koivunen P., Cao S., Backus K.M., Olenchock B.A., Patel H., Zhang Q., Signoretti S., Gerfen G.J., Richardson A.L. (2016). Paracrine induction of HIF by glutamate in breast cancer: EglN1 senses cysteine. Cell.

[272] Song C.W., Lee H., Dings R.P., Williams B., Powers J., Santos T.D., Choi B.H., Park H.J. (2012). Metformin kills and radiosensitizes cancer cells and preferentially kills cancer stem cells. Sci. Rep..

[273] Tang T., Lord J.M., Norman R.J., Yasmin E., Balen A.H. (2012). Insulin-sensitizing drugs (metformin, rosiglitazone, pioglitazone, D-chiro-inositol) for women with polycystic ovary syndrome, oligo amenorrhea and subfertility. Cochrane Database Syst. Rev..

[274] Belli S.H., Graffigna M.N., Oneto A., Otero P., Schurman L., Levalle O.A. (2004). Effect of rosiglitazone on insulin resistance, growth factors, and reproductive disturbances in women with polycystic ovary syndrome. Fertil. Steril..

[275] Morley L.C., Tang T., Yasmin E., Norman R.J., Balen A.H. (2017). Insulin-sensitizing drugs (metformin, rosiglitazone, pioglitazone, D-chiro-inositol) for women with polycystic ovary syndrome, oligo amenorrhea and subfertility. Cochrane Database Syst. Rev..

[276] Bowker S.L., Majumdar S.R., Veugelers P., Johnson J.A. (2006). Increased cancer-related mortality for patients with type 2 diabetes who use sulfonylureas or insulin. Diabetes Care.

[277] Alfarouk K.O., Shayoub M.E., Muddathir A.K., Elhassan G.O., Bashir A.H. (2011). Evolution of tumor metabolism might reflect carcinogenesis as a reverse evolution process (dismantling of multicellularity). Cancers (Basel).

[278] Vomachka A.J., Pratt S.L., Lockefeer J.A., Horseman N.D. (2000). Prolactin gene-disruption arrests mammary gland development and retards T-antigen-induced tumor growth. Oncogene.

[279] Seo E.J., Sugimoto Y., Greten H.J., Efferth T. (2018). Repurposing of bromocriptine for cancer therapy. Front. Pharm..

[280] Nooshinfar E., Safaroghli-Azar A., Bashash D., Akbari M.E. (2017). Melatonin, an inhibitory agent in breast cancer. Breast Cancer.

[281] Hill S.M., Belancio V.P., Dauchy R.T., Xiang S., Brimer S., Mao L., Hauch A., Lundberg P.W., Summers W., Yuan L. (2015). Melatonin: An inhibitor of breast cancer. Endocr. Relat. Cancer.

[282] Sonehara N.M., Lacerda J.Z., Jardim-Perassi B.V., de Paula R., Moschetta-Pinheiro M.G., Souza Y.S.T., de Andrade J.C.J., De Campos Zuccari D.A.P. (2019). Melatonin regulates tumor aggressiveness under acidosis condition in breast cancer cell lines. Oncol. Lett..

[283] Moretti E., Favero G., Rodella L.F., Rezzani R. (2020). Melatonin’s antineoplastic potential against glioblastoma. Cells.

[284] Alvarez-García V., González A., Alonso-González C., Martínez-Campa C., Cos S. (2013). Regulation of vascular endothelial growth factor by melatonin in human breast cancer cells. J. Pineal Res..

[285] Lacerda J.Z., Ferreira L.C., Lopes B.C., Aristizabal-Pachon A.F., Bajgelman M.C., Borin T.F., Zuccari D. (2019). Therapeutic potential of melatonin in the regulation of MiR-148a-3p and angiogenic factors in breast cancer. Microrna.

[286] Jardim-Perassi B.V., Lourenco M.R., Doho G.M., Grigolo I.H., Gelaleti G.B., Ferreira L.C., Borin T.F., Moschetta M.G., Pires de Campos Zuccari D.A. (2016). Melatonin regulates angiogenic factors under hypoxia in breast cancer cell lines. Anticancer Agents Med. Chem..

[287] Korkmaz T., Aygenli F., Emisoglu H., Ozcelik G., Canturk A., Yilmaz S., Ozturk N. (2018). Opposite carcinogenic effects of circadian clock gene *BMAL1*. Sci. Rep..

[288] Reiter R.J., Rosales-Corral S.A., Tan D.X., Acuna-Castroviejo D., Qin L., Yang S.F., Xu K. (2017). Melatonin, a full service anti-cancer agent: Inhibition of initiation, progression and metastasis. Int. J. Mol. Sci..

[289] Menéndez-Menéndez J., Hermida-Prado F., Granda-Díaz R., González A., García-Pedrero J.M., Del-Río-Ibisate N., González-González A., Cos S., Alonso-González C., Martínez-Campa C. (2019). Deciphering the molecular basis of melatonin protective effects on breast cells treated with doxorubicin: TWIST1 a transcription factor involved in EMT and metastasis, a novel target of melatonin. Cancers (Basel).

[290] Sanchez-Sanchez A.M., Antolin I., Puente-Moncada N., Suarez S., Gomez-Lobo M., Rodriguez C., Martin V. (2015). Melatonin cytotoxicity is associated to Warburg effect inhibition in Ewing sarcoma cells. PLoS ONE.

[291] Salvati A., Gigantino V., Nassa G., Mirici Cappa V., Ventola G.M., Cracas D.G.C., Mastrocinque R., Rizzo F., Tarallo R., Weisz A. (2020). Global view of candidate therapeutic target genes in hormone-responsive breast cancer. Int. J. Mol. Sci..

[292] Hasan M., Marzouk M.A., Adhikari S., Wright T.D., Miller B.P., Matossian M.D., Elliott S., Wright M., Alzoubi M., Collins-Burow B.M. (2019). Pharmacological, mechanistic, and pharmacokinetic assessment of novel melatonin-tamoxifen drug conjugates as breast cancer drugs. Mol. Pharm..

[293] Sun R.C., Fadia M., Dahlstrom J.E., Parish C.R., Board P.G., Blackburn A.C. (2010). Reversal of the glycolytic phenotype by dichloroacetate inhibits metastatic breast cancer cell growth in vitro and in vivo. Breast Cancer Res. Treat..

[294] Ohashi T., Akazawa T., Aoki M., Kuze B., Mizuta K., Ito Y., Inoue N. (2013). Dichloroacetate improves immune dysfunction caused by tumor-secreted lactic acid and increases antitumor immunoreactivity. Int. J. Cancer.

[295] Wu K.H., Ho C.T., Chen Z.F., Chen L.C., Whang-Peng J., Lin T.N., Ho Y.S. (2018). The apple polyphenol phloretin inhibits breast cancer cell migration and proliferation via inhibition of signals by type 2 glucose transporter. J. Food Drug Anal..

[296] Meiners C. (2011). Clinical response of metastatic breast cancer to multi-targeted therapeutic approach: A single case report. Cancers (Basel).

[297] Bai F., Yu Z., Gao X., Gong J., Fan L., Liu F. (2019). Simvastatin induces breast cancer cell death through oxidative stress up-regulating miR-140-5p. Aging (Albany NY).

[298] Wang Y.C., Chao T.K., Chang C.C., Yo Y.T., Yu M.H., Lai H.C. (2013). Drug screening identifies niclosamide as an inhibitor of breast cancer stem-like cells. PLoS ONE.

[299] Pronzato P., Amoroso D., Bertelli G., Conte P.F., Cusimano M.P., Ciottoli G.B., Gulisano M., Lionetto R., Rosso R. (1989). Phase II study of lonidamine in metastatic breast cancer. Br. J. Cancer.

[300] Bougnoux P., Hajjaji N., Ferrasson M.N., Giraudeau B., Couet C., Le Floch O. (2009). Improving outcome of chemotherapy of metastatic breast cancer by docosahexaenoic acid: A phase II trial. Br. J. Cancer.

[301] Wannous R., Bon E., Gillet L., Chamouton J., Weber G., Brisson L., Goré J., Bougnoux P., Besson P., Roger S. (2014). Suppression of PPARβ, and DHA treatment, inhibit NaV1. 5 and NHE-1 pro-invasive activities. Pflug. Arch..

[302] Li Y., Li P.K., Roberts M.J., Arend R.C., Samant R.S., Buchsbaum D.J. (2014). Multi-targeted therapy of cancer by niclosamide: A new application for an old drug. Cancer Lett..

[303] Naujokat C., Steinhart R. (2012). Salinomycin as a drug for targeting human cancer stem cells. J. Biomed. Biotechnol..

[304] Dinic J., Efferth T., Garcia-Sosa A.T., Grahovac J., Padron J.M., Pajeva I., Rizzolio F., Saponara S., Spengler G., Tsakovska I. (2020). Repurposing old drugs to fight multidrug resistant cancers. Drug Resist. Updat..

[305] Jin W., Li Q., Lin Y., Lu Y., Li H., Wang L., Hu R., Ma L., Wang J., Pang T. (2011). Reversal of imatinib resistance in *BCR-ABL*-positive leukemia after inhibition of the Na^+^/H^+^ exchanger. Cancer Lett..

[306] Li Q.H., Lu Y., Jin W.N., Lin Y.N., Hu R.H., Zhu X.F., Wang J.X., Pang T.X. (2009). Effect of intracellular acidification on drug resistance of leukemia cells with high P-glycoprotein expression. Zhonghua Xue Ye Xue Za Zhi.

[307] Harguindey S., Arranz J.L., Polo Orozco J.D., Rauch C., Fais S., Cardone R.A., Reshkin S.J. (2013). Cariporide and other new and powerful NHE1 inhibitors as potentially selective anticancer drugs—An integral molecular/biochemical/metabolic/clinical approach after one hundred years of cancer research. J. Transl. Med..

[308] Harguindey S., Cragoe E.J. (1992). The Na^+^/H^+^ antiporter in oncology in the light of the spontaneous regression of cancer and cell metabolism. Med. Hypotheses.

[309] Di Sario A., Bendia E., Omenetti A., De Minicis S., Marzioni M., Kleemann H.W., Candelaresi C., Saccomanno S., Alpini G., Benedetti A. (2007). Selective inhibition of ion transport mechanisms regulating intracellular pH reduces proliferation and induces apoptosis in cholangiocarcinoma cells. Dig. Liver Dis..

[310] Luciani F., Spada M., De Milito A., Molinari A., Rivoltini L., Montinaro A., Marra M., Lugini L., Logozzi M., Lozupone F. (2004). Effect of proton pump inhibitor pretreatment on resistance of solid tumors to cytotoxic drugs. J. Natl. Cancer Inst..

[311] Lee Z.W., Teo X.Y., Song Z.J., Nin D.S., Novera W., Choo B.A., Dymock B.W., Moore P.K., Huang R.Y., Deng L.W. (2017). Intracellular hyper-acidification potentiated by hydrogen sulfide mediates invasive and therapy resistant cancer cell death. Front. Pharm..

[312] Kulshrestha A., Katara G.K., Ibrahim S.A., Riehl V., Sahoo M., Dolan J., Meinke K.W., Pins M.R., Beaman K.D. (2019). Targeting V-ATPase isoform restores cisplatin activity in resistant ovarian cancer: Inhibition of autophagy, endosome function, and ERK/MEK pathway. J. Oncol..

[313] Vernimmen F.J., Slabbert J.P., Wilson J.A., Fredericks S., Melvill R. (2005). Stereotactic proton beam therapy for intracranial arteriovenous malformations. Int. J. Radiat. Oncol. Biol. Phys..

[314] Newhauser W.D., Zhang R. (2015). The physics of proton therapy. Phys. Med. Biol..

[315] Janku F., McConkey D.J., Hong D.S., Kurzrock R. (2011). Autophagy as a target for anticancer therapy. Nat. Rev. Clin. Oncol..

[316] Fais S., Overholtzer M. (2018). Cell-in-cell phenomena in cancer. Nat. Rev. Cancer.

[317] Verbaanderd C., Maes H., Schaaf M.B., Sukhatme V.P., Pantziarka P., Sukhatme V., Agostinis P., Bouche G. (2017). Repurposing drugs in oncology (ReDO)-chloroquine and hydroxychloroquine as anti-cancer agents. Ecancermedicalscience.

[318] Briceño E., Reyes S., Sotelo J. (2003). Therapy of glioblastoma multiforme improved by the antimutagenic chloroquine. Neurosurg. Focus.

[319] Pellegrini P., Strambi A., Zipoli C., Hagg-Olofsson M., Buoncervello M., Linder S., De Milito A. (2014). Acidic extracellular pH neutralizes the autophagy-inhibiting activity of chloroquine: Implications for cancer therapies. Autophagy.

[320] Saliba K.J., Kirk K. (1999). pH regulation in the intracellular malaria parasite, Plasmodium falciparum. H^+^ extrusion via a V-type H^+^-ATPase. J. Biol. Chem..

[321] Moriyama Y., Hayashi M., Yatsushiro S., Yamamoto A. (2003). Vacuolar proton pumps in malaria parasite cells. J. Bioenerg. Biomembr..

[322] Wang M., Cao R., Zhang L., Yang X., Liu J., Xu M., Shi Z., Hu Z., Zhong W., Xiao G. (2020). Remdesivir and chloroquine effectively inhibit the recently emerged novel coronavirus (2019-nCoV) in vitro. Cell Res..

[323] Vincent M.J., Bergeron E., Benjannet S., Erickson B.R., Rollin P.E., Ksiazek T.G., Seidah N.G., Nichol S.T. (2005). Chloroquine is a potent inhibitor of SARS coronavirus infection and spread. Virol. J..

[324] Harguindey S., Galdós I., Takita H. (1994). Lung cancer as a scar tumor: Apropos a case and the possible etiopathogenetic mechanisms. Med. Interna.

[325] Samanta D., Gilkes D.M., Chaturvedi P., Xiang L., Semenza G.L. (2014). Hypoxia-inducible factors are required for chemotherapy resistance of breast cancer stem cells. Proc. Natl. Acad. Sci. USA.

[326] Semenza G.L. (2003). Targeting HIF-1 for cancer therapy. Nat. Rev. Cancer.

[327] Xiang L., Gilkes D.M., Chaturvedi P., Luo W., Hu H., Takano N., Liang H., Semenza G.L. (2014). Ganetespib blocks HIF-1 activity and inhibits tumor growth, vascularization, stem cell maintenance, invasion, and metastasis in orthotopic mouse models of triple-negative breast cancer. J. Mol. Med. (Berl.).

[328] Pouyssegur J., Dayan F., Mazure N.M. (2006). Hypoxia signaling in cancer and approaches to enforce tumour regression. Nature.

[329] Semenza G.L. (2012). Hypoxia-inducible factors: Mediators of cancer progression and targets for cancer therapy. Trends Pharm. Sci..

[330] Hsu C.-W., Huang R., Khuc T., Shou D., Bullock J., Grooby S., Griffin S., Zou C., Little A., Astley H. (2016). Identification of approved and investigational drugs that inhibit hypoxia-inducible factor-1 signaling. Oncotarget.

[331] Wigerup C., Pahlman S., Bexell D. (2016). Therapeutic targeting of hypoxia and hypoxia-inducible factors in cancer. Pharm. Ther..

[332] Zhou L., Xu S., Yin W., Lin Y., Du Y., Jiang Y., Wang Y., Zhang J., Wu Z., Lu J. (2017). Weekly paclitaxel and cisplatin as neoadjuvant chemotherapy with locally advanced breast cancer: A prospective, single arm, phase II study. Oncotarget.

[333] Sledge G.W. (1995). Doxorubicin/paclitaxel combination chemotherapy for metastatic breast cancer: The eastern cooperative oncology group experience. Semin. Oncol..

[334] Kubo M. (2020). Adjuvant endocrine treatment for estrogen receptor (ER)-positive/*HER2*-negative breast cancer. Chin. Clin. Oncol..

[335] Pineda-Moncusí M., Garcia-Giralt N., Diez-Perez A., Tusquets I., Servitja S., Albanell J., Prieto-Alhambra D., Nogués X. (2020). Thromboembolic, cardiovascular and overall mortality risks of aromatase inhibitors, compared with tamoxifen treatment: An outpatient-register-based retrospective cohort study. Adv. Med. Oncol..

[336] Kharb R., Haider K., Neha K., Yar M.S. (2020). Aromatase inhibitors: Role in postmenopausal breast cancer. Arch. Pharm..

[337] Takahashi M., Masuda N., Nishimura R., Inoue K., Ohno S., Iwata H., Hashigaki S., Muramatsu Y., Umeyama Y., Toi M. (2020). Palbociclib-letrozole as first-line treatment for advanced breast cancer: Updated results from a Japanese phase 2 study. Cancer Med..

[338] Schwartz L., Seyfried T., Alfarouk K.O., Da Veiga Moreira J., Fais S. (2017). Out of Warburg effect: An effective cancer treatment targeting the tumor specific metabolism and dysregulated pH. Semin. Cancer Biol..

[339] Pelicano H., Martin D.S., Xu R.H., Huang P. (2006). Glycolysis inhibition for anticancer treatment. Oncogene.

[340] Zhang H. (2017). Will cancer cells be defeated by sodium bicarbonate?. Sci. China Life Sci..

[341] Konishi N., Nishii K., Hayashi I., Nakaoka S., Matsumoto K., Yabuno T., Kitahori Y., Hiasa Y. (1993). Inhibitory effect of potassium citrate on rat renal tumors induced by N-Ethyl-N-hydroxyethylnitrosamine followed by potassium dibasic phosphate. Jpn. J. Cancer Res..

[342] Jamrozik S.I., Bennet A.P., James-Deidier A., Tremollieres F., Saint-Martin F., Dumoulin S., Valat-Coustols M., de Glisezinski I., Tremoulet M., Manelfe C. (1996). Treatment with long acting repeatable bromocriptine (Parlodel-LAR*) in patients with macroprolactinomas: Long-term study in 29 patients. J. Endocrinol. Investig..

[343] Ferrari C., Barbieri C., Caldara R., Mucci M., Codecasa F., Paracchi A., Romano C., Boghen M., Dubini A. (1986). Long-lasting prolactin-lowering effect of cabergoline, a new dopamine agonist, in hyperprolactinemic patients. J. Clin. Endocrinol. Metab..

[344] Papantoniou K., Castano-Vinyals G., Espinosa A., Aragones N., Perez-Gomez B., Ardanaz E., Altzibar J.M., Sanchez V.M., Gomez-Acebo I., Llorca J. (2016). Breast cancer risk and night shift work in a case-control study in a Spanish population. Eur. J. Epidemiol..

[345] Cordina-Duverger E., Menegaux F., Popa A., Rabstein S., Harth V., Pesch B., Bruning T., Fritschi L., Glass D.C., Heyworth J.S. (2018). Night shift work and breast cancer: A pooled analysis of population-based case-control studies with complete work history. Eur. J. Epidemiol..

[346] Bustamante-Montes L.P., Flores-Meza B., Hernández-Valero M.A., Cárdenas-López A., Dolores-Velázquez R., Borja-Bustamante P., Borja-Aburto V.H. (2019). Night shift work and risk of breast cancer in women. Arch. Med. Res..

[347] Coleman M.P., Reiter R.J. (1992). Breast cancer, blindness and melatonin. Eur. J. Cancer.

[348] Barchas J., DaCosta F., Spector S. (1967). Acute pharmacology of melatonin. Nature.

[349] Silman R.E. (1993). Melatonin: A contraceptive for the nineties. Eur. J. Obs. Gynecol. Reprod. Biol..

[350] Devesa J., Nunez I., Agra C., Bejarano A., Devesa P. (2018). Treatment with growth hormone (GH) increased the metabolic activity of the brain in an elder patient, not GH-deficient, who suffered mild cognitive alterations and had an ApoE 4/3 genotype. Int. J. Mol. Sci..

[351] Weishaupt J.H., Bartels C., Polking E., Dietrich J., Rohde G., Poeggeler B., Mertens N., Sperling S., Bohn M., Huther G. (2006). Reduced oxidative damage in ALS by high-dose enteral melatonin treatment. J. Pineal Res..

[352] Reiter R.J., Sharma R., Ma Q., Rorsales-Corral S., de Almeida Chuffa L.G. (2020). Melatonin inhibits Warburg-dependent cancer by redirecting glucose oxidation to the mitochondria: A mechanistic hypothesis. Cell Mol. Life Sci..

[353] Abdel Moneim A.E., Guerra-Librero A., Florido J., Shen Y.Q., Fernandez-Gil B., Acuna-Castroviejo D., Escames G. (2017). Oral mucositis: Melatonin gel an effective new treatment. Int. J. Mol. Sci..

[354] Rusanova I., Martinez-Ruiz L., Florido J., Rodriguez-Santana C., Guerra-Librero A., Acuna-Castroviejo D., Escames G. (2019). Protective effects of melatonin on the skin: Future perspectives. Int. J. Mol. Sci..

[355] Masoud G.N., Li W. (2015). HIF-1α pathway: Role, regulation and intervention for cancer therapy. Acta Pharm. Sin. B.

[356] Pastorek J., Pastorekova S. (2015). Hypoxia-induced carbonic anhydrase IX as a target for cancer therapy: From biology to clinical use. Semin. Cancer Biol..

[357] Evans R.J. (1972). Acid-base changes in patients with intractable pain and malignancy. Can. J. Surg..

[358] Hamaguchi R., Narui R., Wada H. (2020). Effects of alkalization therapy on chemotherapy outcomes in metastatic or recurrent pancreatic cancer. Anticancer Res..

[359] Hoang B.X., Le B.T., Tran H.D., Hoang C., Tran H.Q., Tran D.M., Pham C.Q., Pham T.D., Ha T.V., Bui N.T. (2011). Dimethyl sulfoxide–sodium bicarbonate infusion for palliative care and pain relief in patients with metastatic prostate cancer. J. Pain Palliat. Care Pharm..

[360] Salim A.S. (1992). Oxygen-derived free-radical scavengers prolong survival in colonic cancer. Chemotherapy.

[361] Voss N.C.S., Dreyer T., Henningsen M.B., Vahl P., Honore B., Boedtkjer E. (2020). Targeting the acidic tumor microenvironment: Unexpected pro-neoplastic effects of oral NaHCO_3_ therapy in murine breast tissue. Cancers (Basel).

[362] Goldsmith D.J., Forni L.G., Hilton P.J. (1997). Bicarbonate therapy and intracellular acidosis. Clin. Sci. (Lond.).

[363] Atwal K.S., O’Neil S.V., Ahmad S., Doweyko L., Kirby M., Dorso C.R., Chandrasena G., Chen B.C., Zhao R., Zahler R. (2006). Synthesis and biological activity of 5-aryl-4-(4-(5-methyl-1H-imidazol-4-yl)piperidin-1-yl)pyrimidine analogs as potent, highly selective, and orally bioavailable NHE-1 inhibitors. Bioorg. Med. Chem. Lett..

[364] Kohno K., Miyake M., Sano O., Tanaka-Kataoka M., Yamamoto S., Koya-Miyata S., Arai N., Fujii M., Watanabe H., Ushio S. (2008). Anti-inflammatory and immunomodulatory properties of 2-amino-3H-phenoxazin-3-one. Biol. Pharm. Bull..

